# Quinuclidin-3-one-based quaternary ammonium compounds as potent antibacterials against *Staphylococcus aureus* and *Listeria monocytogenes*

**DOI:** 10.17305/bb.2026.14037

**Published:** 2026-04-22

**Authors:** Antonio Sabljić, Doris Čarija, Karolina Begić, Renata Odžak, Rozelindra Čož-Rakovac, Josip Tomaš, Matilda Šprung

**Affiliations:** 1 Department of Chemistry, Faculty of Science, University of Split, Split, Croatia; 2 Ruder Bošković Institute, Laboratory for Biotechnology in Aquaculture, Division of Materials Chemistry, Zagreb, Croatia

**Keywords:** Quaternary ammonium compounds, QACs, quinuclidin-3-one QACs, bacterial resistance, cytotoxicity, zebrafish embryotoxicity, critical micelle concentration, cmc, mode of action

## Abstract

Bacterial resistance and the cytotoxicity of conventional quaternary ammonium compounds (QACs) highlight the necessity for safer and more effective membrane-active antibacterials. This study aimed to synthesize a homologous series of quinuclidin-3-one-based QACs (QO-C_12_, QO-C_14_, and QO-C_16_) and to elucidate their structure–activity relationships through comprehensive antibacterial, antibiofilm, cytotoxicity, embryotoxicity, mechanistic, and physicochemical analyses. The compounds were evaluated against clinically relevant pathogens using broth microdilution, growth and time-kill assays, biofilm inhibition testing, membrane permeabilization analysis, cytotoxicity testing in human retinal pigment epithelial (RPE1) and human embryonic kidney (HEK293) cells, zebrafish embryotoxicity assessments, and critical micelle concentration (CMC) measurements. Antibacterial potency was found to increase with alkyl chain length, with QO-C_16_ exhibiting the highest activity, including minimum inhibitory concentrations (MICs) of 8 µM against *Staphylococcus aureus* (including methicillin-resistant strains) and 4 µM against *Listeria monocytogenes*. In Dulbecco’s modified Eagle medium (DMEM), MIC values were up to 32-fold lower than those observed in broth media, indicating a significant medium-dependent effect. QO-C_14_ and QO-C_16_ demonstrated robust antibiofilm activity, while QO-C_16_ produced the fastest bactericidal and membrane-disruptive effects. The critical micelle concentration decreased with increasing chain length, aligning with enhanced hydrophobicity and membrane affinity. Compared to commercial QACs, the novel compounds exhibited lower cytotoxicity in human cell lines; however, zebrafish embryotoxicity did increase with chain length. Overall, quinuclidin-3-one represents a promising scaffold for the development of QACs, with QO-C_16_ identified as the lead compound for further optimization toward selective antibacterial agents.

## Introduction

The emergence of bacterial resistance represents a critical global health challenge, primarily attributed to the excessive and uncontrolled utilization of antimicrobial agents, including disinfectants and antiseptics [1]. This situation has catalyzed efforts to develop novel antimicrobials that maintain effectiveness while minimizing the selection pressure that fosters resistance in pathogenic bacteria [2].

Quaternary ammonium compounds (QACs) are cationic surfactants characterized by a positively charged nitrogen atom bonded to four identical or diverse substituents, such as alkyl or aryl groups. Based on the number of positively charged nitrogen atoms, QACs can be categorized as mono-, bis-, and polyvalent compounds. Mono- and bis-QACs typically exhibit the most significant antimicrobial activity; however, certain tris- and tetracationic derivatives have also shown remarkable antimicrobial potency [3, 4]. Chemically, QACs are amphipathic molecules with both hydrophilic and hydrophobic regions, which influence their physicochemical properties and, consequently, their antimicrobial efficacy [5]. A crucial characteristic of surfactants like QACs is their capacity to self-assemble into micelles, defined by the critical micelle concentration (*cmc*), which is vital for understanding their surface activity and performance in various applications.

The antimicrobial mechanism of QACs is analogous to that of detergents, disrupting bacterial membranes through a two-step process. Initially, the positively charged nitrogen atom engages electrostatically with negatively charged components of the bacterial membrane. Subsequently, the hydrophobic portion integrates into the lipid bilayer, resulting in membrane destabilization, cytoplasmic leakage, and ultimately cell death [6–10]. Structure–activity relationship (SAR) studies reveal that substituents on the quaternary nitrogen atom significantly influence antimicrobial activity by modulating electrostatic interactions and hydrogen-bonding capacities. Hydroxyl and amino groups can act as both hydrogen-bond donors and acceptors, while carbonyl groups function solely as hydrogen-bond acceptors. Collectively, these characteristics can enhance noncovalent interactions with bacterial targets, including membrane lipids, proteins, and nucleic acids [11]. QACs containing polar functional groups typically exhibit increased water solubility, thereby enhancing their availability and efficacy in aqueous bacterial environments [12]. Furthermore, their membranolytic action is influenced by the hydrophilic-to-hydrophobic ratio, indicating that achieving an optimal balance is crucial for maximizing antimicrobial outcomes [1].

Despite their antimicrobial potential, QACs also demonstrate significant cytotoxicity to mammalian cells, particularly affecting respiratory and reproductive tissues [13]. Additionally, their chemical stability and prevalent usage result in environmental accumulation, posing ecological risks, especially in aquatic ecosystems [14]. In such environments, sublethal concentrations of QACs can contribute to bacterial resistance, especially in regions with high industrial application [7]. In light of their cytotoxicity and potential to drive bacterial resistance, current research is focused on designing QACs with enhanced selectivity, reduced environmental persistence, and minimized resistance potential [15].

Natural products have emerged as promising frameworks for the design of new QACs that may mitigate resistance development while minimizing adverse effects on human health and the environment [16–18]. Quinuclidine, a rigid aliphatic bicyclic amine featuring a bridgehead nitrogen atom, is present in several biologically active compounds, including quinine, which has been utilized in the treatment of malaria and cardiac arrhythmias [19, 20]. In our prior research, quinuclidine-derived QACs were identified as promising antimicrobial agents, exhibiting tunable biological properties. The antimicrobial efficacy of these compounds, derived from variously substituted quinuclidine derivatives such as 3-aminoquinuclidine, quinuclidin-3-ol, and 3-chloroquinuclidine with long alkyl chains, was evaluated against a range of Gram-positive and Gram-negative bacteria, confirming their potential as novel antibacterial agents [21–23]. Building on these findings, we focused on quinuclidin-3-one as a novel scaffold for QAC design. Its carbonyl functionality at the C-3 position imparts distinct hydrogen-bonding properties and polarity compared to previously studied derivatives, potentially enhancing interactions with bacterial membranes while maintaining an optimal amphiphilic balance through the hydrophobic character of long alkyl chains. This rationale guided the design of quinuclidin-3-one-based quaternary ammonium compounds (QO-QACs), aimed at improving antibacterial potency and selectivity. The biological activity of these QACs was significantly influenced by the nature of the substituent at the C-3 position of the quinuclidine ring [24]. The introduction of longer alkyl chains (C14–C16) markedly enhanced antibacterial activity, underscoring the importance of hydrophobic interactions in their mode of action.

In this study, we aimed to synthesize and evaluate a homologous series of quinuclidin-3-one-based QACs (QO-C_1__2_, QO-C_1__4_, and QO-C_16_) varying in alkyl chain length. We assessed their antibacterial and antibiofilm activity against clinically relevant pathogens, bactericidal kinetics, susceptibility to efflux-mediated resistance, cytotoxicity in human cell lines, *in vivo* embryotoxicity in zebrafish, and key physicochemical properties, thereby establishing structure–activity relationships to guide the development of safer QAC-based antimicrobials.

## Materials and methods

### Synthesis of quaternary ammonium compounds from quinuclidin-3-one

Quinuclidin-3-one was synthesized in high yields (*η*>90%) by treating commercially available quinuclidin-3-one hydrochloride (200 mg, 1.24 mmol) (Sigma Aldrich, 97%) with a saturated aqueous solution of potassium hydroxide (7.13 mmol in 0.4 mL H_2_O). The reaction mixture was extracted with chloroform (10×2 mL). The combined organic extracts were dried over anhydrous potassium carbonate (K_2_CO_3_), filtered, and concentrated under reduced pressure. The resulting product was stored under reduced pressure over anhydrous calcium chloride (CaCl_2_) to ensure desiccation. The obtained pure quinuclidin-3-one served as a precursor for the synthesis of new quaternary ammonium compounds.

In the quaternization reaction of quinuclidin-3-one, the corresponding quaternization reagents were employed: 1-bromododecane (Alfa Aesar, 98%), 1-bromotetradecane (Alfa Aesar, 98%), and 1-bromohexadecane (Alfa Aesar, 98%) in dry acetone (≥ 99.5%). Thin-layer chromatography (TLC) was performed using DC-Alufolien Aluminiumoxid 60 F254 (Merck) in the appropriate solvent system (MeOH:CHCl3/1:5). Dye detection after plate development was achieved through a reversible reaction with iodine vapor. The melting points of the QACs were determined in open capillaries using a Büchi B-540 apparatus.

Proton nuclear magnetic resonance (^1^H NMR) and carbon-13 nuclear magnetic resonance (^13^C NMR) spectra of the newly synthesized compounds were recorded in CDCl3 solution using a Bruker AV600 spectrometer (150 and 600 MHz) at room temperature (Supplementary information, ** Figures S1**-**S9**). Mass spectra were collected on an liquid chromatography/mass spectrometry (LC/MS) quadrupole time-of-flight (QTOF) system, utilizing the Sciex TripleTOF 6600+ instrument for high-resolution analysis.

***N*-Dodecyl-3-oxoquinuclidinium bromide** (QO-C12): Equimolar amounts of quinuclidin-3-one (0.4 mmol) and 1-bromododecane were reacted in dry acetone under an inert nitrogen atmosphere at room temperature, with continuous stirring for 24 h, following a previously reported quaternization procedure [23]. The progress of the chemical reactions was monitored by TLC. Upon completion, the reaction mixture was concentrated under reduced pressure using a rotary evaporator. The reaction product was purified by recrystallization from diethyl ether to isolate the reaction product as a white powder with high spectral purity, as confirmed by 1H NMR, 13C NMR, and high-resolution mass spectrometry (HRMS) analysis (*η* ═ 92%), *m.p.* 236–238 ^∘^C.

^1^H NMR (600 MHz, CDCl3) *δ* (ppm) ═ 4.68 (s, 2H, H-2), 4.15–3.98 (m, 4H, H-6, H-8), 3.79–3.73 (m, 2H, H-1’), 2.87–2.81 (m, 1H, H-4), 2.49–2.41 (m, 2H, H-7), 2.40–2.32 (m, 2H, H-5), 1.85–1.71 (m, 2H, H-2’), 1.45–1.37 (m, 4H, H-3’, H-4’), 1.36 (s, 14H, H-5’ – H-11’), 0.89 (t, 3_J_ = 7.01 Hz, 3H, H-12’). 13C NMR (150 MHz, CDCl3) *δ* (ppm) ═ 201.3 (C=O), 65.9 (C-2), 64.4 (C-1), 54.8 (C-6, C-8), 37.2 (C-4), 31.9 (C-2’), 29.6 (C-3’, C-4’), 29.5 (C-5’), 29.4 (C-6’), 29.3 (C-7’), 29.2 (C-8’), 26.4 (C-9’), 22.7 (C-10’), 22.6 (C-11’), 21.3 (C-5, C-7), 14.2 (C-12’). HRMS (ESI+): *m/z* calculated for C19H36NO+ ([M]+) 294.2791, found 294.2790, err [ppm] 0.34.

***N*-Tetradecyl-3-oxoquinuclidinium bromide** (QO-C14): Equimolar amounts of quinuclidin-3-one (0.4 mmol) and 1-bromotetradecane were reacted under the same conditions as previously described. The reaction product was obtained as a white powder with high spectral purity, as confirmed by 1H NMR, 13C NMR, and HRMS analysis (*η* ═ 98%), *m.p.* 243–245 ^∘^C.

^1^H NMR (600 MHz, CDCl3) *δ* (ppm) ═ 4.68 (s, 2H, H-2), 4.11–4.07 (m, 2H, H-8), 4.02–3.98 (m, 2H, H-6), 3.76–3.73 (m, 2H, H-1’), 2.81 (quint, 3_J_ = 3.01 Hz, 1H, H-4), 2.47–2.45 (m, 2H, H-7), 2.43–2.36 (m, 2H, H-5), 1.88–1.78 (m, 2H, H-2’), 1.39–1.31 (m, 4H, H-3’, H-4’), 1.29 (s, 18H, H-5’ – H-13’), 0.88 (t, 3_J_ = 7.03 Hz, 3H, H-14’). 13C NMR (150 MHz, CDCl3) *δ* (ppm) ═ 201.3 (C=O), 66.1 (C-2), 64.6 (C-1’), 54.9 (C-6, C-8), 37.4 (C-4), 32.1 (C-2’), 29.9 (C-3’), 29.8 (C-4’, C-5’), 29.8 (C-6’), 29.7 (C-7’), 29.6 (C-8’), 29.6 (C-9’), 29.4 (C-10’), 26.6 (C-11’), 22.9 (C-12’), 22.8 (C-13’), 21.5 (C-5, C-7), 14.3 (C-14’). HRMS (ESI+): *m/z* calculated for C21H40NO+ ([M]+) 322.3104, found 322.3102, err [ppm] 0.62.

***N*-Hexadecyl-3-oxoquinuclidinium bromide **(QO-C16): Equimolar amounts of quinuclidin-3-one (0.4 mmol) and 1-bromohexadecane were reacted under the same conditions as previously detailed. The reaction product was obtained as a white powder with high spectral purity, as confirmed by 1H NMR, 13C NMR, and HRMS analysis (*η* ═ 98%), *m.p.* 252–254 ^∘^C.

^1^H NMR (600 MHz, CDCl3) *δ* (ppm) ═ 4.70 (s, 2H, H-2), 4.15–4.09 (m, 2H, H-8), 4.03–3.95 (m, 2H, H-6), 3.77–3.71 (m, 2H, H-1’), 2.82 (quint, 3_J_ = 3.02 Hz, 1H, H-4), 2.49–2.42 (m, 2H, H-7), 2.40–2.30 (m, 2H, H-5), 1.87–1.76 (m, 2H, H-2’), 1.42–1.31 (m, 4H, H-3’, H-4’), 1.28 (s, 22H, H-5’ – H-15’), 0.89 (t, 3_J_ = 7.03 Hz, 3H, H-16’). 13C NMR (150 MHz, CDCl3) *δ* (ppm) ═ 201.0 (C=O), 65.9 (C-2), 64.4 (C-1’), 54.7 (C-6, C-8), 37.2 (C-4), 31.9 (C-2’), 29.8 (C-3’), 29.7 (C-4’, C-5’), 29.6 (C-6’), 29.5 (C-7’), 29.4 (C-8’, C-9’, C-10’), 29.3 (C-11’), 29.2 (C-12’), 26.4 (C-13’), 22.7 (C-14’), 22.6 (C-15’), 21.3 (C-5, C-7), 14.2 (C-16’). HRMS (ESI+): *m/z* calculated for C23H44NO+ ([M]+) 350.3417, found 350.3409, err [ppm] 2.28.

### Antibacterial activity

The biological activity of the synthesized quinuclidin-3-one quaternary ammonium compounds was evaluated against various Gram-positive (*Staphylococcus aureus* American Type Culture Collection (ATCC) 25923, *Staphylococcus aureus* MRSA (clinical isolate, University Hospital Split), *Staphylococcus aureus* ATCC 33591, *Bacillus cereus* ATCC 14579, *Listeria monocytogenes* ATCC 7644, and *Enterococcus faecalis* ATCC 29212) and Gram-negative (*Escherichia coli* ATCC 25922, *Salmonella enterica* (food isolate of unknown origin, maintained in the culture collection of the Department of Chemistry, Faculty of Science, University of Split), and *Pseudomonas aeruginosa* ATCC 27853) bacterial strains. The broth microdilution test was conducted in accordance with the “*Methods for Dilution Antimicrobial Susceptibility Test for Bacteria That Grow Aerobically*” [25] of the Clinical and Laboratory Standards Institute. Fresh Mueller-Hinton broth (Biolife, Graz, Austria) was inoculated with the selected strain and incubated overnight at either 35 ^∘^C or 37 ^∘^C with shaking at 220 rpm. The following day, the culture was diluted in the selected medium and incubated under the same conditions until reaching the exponential growth phase (OD600 = 0.3–0.5). Using a previously established OD600-CFU/mL calibration curve, the culture was diluted to a concentration of 5 × 10^5^ CFU/mL. This cell concentration was then added to wells of 96-microtiter plates containing a twofold dilution series of the synthesized compounds, prepared over a concentration range of 250–0.25 µM. The microtiter plates were incubated overnight at either 35 ^∘^C or 37 ^∘^C, depending on the bacterial strain. The minimum inhibitory concentration (MIC) values were visually determined and confirmed by adding 20 µL of 2-(4-iodophenyl)-3-(4-nitrophenyl)-5-phenyltetrazolium chloride (INT; 6 mg/mL), which is reduced to a red formazan by metabolically active bacterial cells.

### Antibacterial activity in different nutrient media

*Staphylococcus aureus* ATCC 25923 was selected as the representative Gram-positive bacterium. An overnight culture was prepared in Mueller-Hinton broth (MHB) at 37 ^∘^C with constant shaking at 220 rpm. The next day, the bacterial culture was diluted in temperature-controlled MHB and grown to the exponential phase (OD600 = 0.3–0.5). For MIC determination in cation-adjusted MHB (CA-MHB), the culture was diluted to a final inoculum of 5 × 10^5^ CFU/mL in CA-MHB containing 85 mM Na^+^ and 10 mM Mg^2+^. For MIC determination in Dulbecco’s modified Eagle medium (DMEM, Capricorn Scientific), the exponential-phase cells were collected by centrifugation at 4000 × g at room temperature and resuspended in DMEM without fetal bovine serum or other protein supplements, maintaining the same final concentration of 5 × 10^5^ CFU/mL. The pH of each medium was verified to be within the range of 7.2 - 7.4 prior to inoculation, and bacterial viability in CA-MHB and DMEM was confirmed using untreated growth controls.

Antibacterial activity was assessed using the broth microdilution method in 96-well microplates. Twofold serial dilutions of the tested compounds were prepared in the corresponding medium as described above. Aliquots of 50 µL of the bacterial suspension were added to each well, and the plates were incubated overnight at 37 ^∘^C under ambient conditions. The MIC was defined as the lowest compound concentration at which no visible bacterial growth was observed. For assays performed in CA-MHB, visual MIC readings were further confirmed by adding INT (6 mg/mL), as previously described.

### Potential for bacterial resistance development

The potential for bacterial resistance development was assessed by determining the MIC in the presence of the protonophore and ATP synthesis inhibitor carbonyl cyanide-3-chlorophenylhydrazone (CCCP). CCCP disrupts the proton motive force across the bacterial cytoplasmic membrane, thereby inhibiting ATP synthesis and impairing the activity of ATP-dependent efflux pumps. If the MIC of a tested compound remains unchanged in the presence of CCCP, it suggests that the compound is not a substrate for ATP-dependent efflux pumps, indicating low susceptibility to efflux-mediated resistance under the tested conditions. The bacterial strain used in this experiment was *Staphylococcus aureus* ATCC 33591 (MRSA), which harbors genes encoding the Qac efflux pumps. The overnight culture was diluted in fresh MHB and cultured in an orbital shaker at 37 ^∘^C and 220 rpm until reaching the exponential growth phase (OD600 = 0.3–0.5). The culture was then diluted to a final concentration of 5 × 10^5^ CFU/mL in MHB supplemented with 10 µM CCCP. Twofold serial dilutions of the tested compounds were prepared in MHB supplemented with 10 µM CCCP in a 96-well plate. A total of 50 µL of the prepared cell suspension was pipetted into the plate containing the appropriate concentrations of the tested compounds. The microtiter plate was incubated overnight at 37 ^∘^C, after which MIC values were determined visually.

### Time-resolved growth analysis

The time-resolved growth analysis was conducted using *Listeria monocytogenes* ATCC 7644, the bacterial strain exhibiting the highest antibacterial activity against the tested compounds. An overnight culture of the corresponding bacterial strain was diluted in fresh temperature-controlled MHB and incubated in a shaker at 37 ^∘^C and 220 rpm. After reaching the exponential growth phase (OD600 = 0.3–0.5), the culture was diluted to a final concentration of 5 × 10^4^ CFU/mL. This diluted culture (50 µL) was added to wells of a microtiter plate containing no antimicrobial agent (untreated control) and to wells with the tested compounds at 1/2 MIC and MIC concentrations. The growth of bacterial cultures was monitored spectrophotometrically for approximately 11 h at 10-minute intervals at 595 nm using a microplate reader (Tecan Infinite 200 PRO fluorometer). The experiment was performed in three independent biological replicates, each in triplicate wells (*n* ═ 3). Results were compared to the untreated control (positive control, PC) and the sterile MHB-only negative control (NC). Growth parameters were derived from background-corrected optical density (OD) values and included area under the curve (AUC, calculated via trapezoidal integration), lag phase (defined as the time required to exceed baseline + 3× standard deviation of the first five time points, sustained for ≥3 consecutive readings), maximum growth rate (ΔOD/h, calculated from the steepest 30-minute sliding window), and growth amplitude (maximum OD − baseline OD). Growth inhibition (%) was calculated as 100 × (1 – AUC_treated / AUC_control). Results are presented as mean ± standard deviation of three independent biological replicates.

### Biofilm inhibition assay

The antibiofilm activity of the tested compounds was evaluated using *Listeria monocytogenes* ATCC 7644. An overnight bacterial culture was diluted in fresh temperature-controlled MHB and incubated in a shaker at 37 ^∘^C and 220 rpm. Upon reaching the exponential growth phase (OD600 = 0.3–0.5), the culture was adjusted to a cell concentration of 4 × 10^6^ CFU/mL. Aliquots of 50 µL of the prepared culture were dispensed into a 96-well microtiter plate containing the test compounds at final concentrations of 100, 50, 25, and 12.5 µg/mL, prepared through twofold serial dilution. The microtiter plate was incubated for 24 h at 37 ^∘^C. After incubation, the culture medium was aspirated from the wells, taking care not to disturb any biofilm that may have formed. The wells were washed with sterile ultrapure Milli-Q water, which was subsequently aspirated. To fix the biofilm, the microtiter plate was allowed to dry for one hour at 60 ^∘^C. After drying, 100 µL of a 0.1% crystal violet solution was added to each well and incubated for one hour at room temperature. Unbound dye was removed by rinsing with sterile ultrapure Milli-Q water, and the plate was dried for 30 min at room temperature. The crystal violet bound to the biofilm was solubilized by adding 100 µL of 70% ethanol and incubating for 30 min. The solution was transferred to a new microtiter plate, and absorbance was measured at 595 nm using a Tecan Infinite 200 PRO plate reader. Untreated cells served as the positive control (maximum biofilm formation), and sterile MHB served as the negative control. The percentage of biofilm inhibition relative to the untreated control was calculated as follows:

% Biofilm Formed ═ (OD_treated / OD_control) × 100

% Biofilm Inhibition = 100 − % Biofilm Formed

where OD_treated is the absorbance of wells containing the test compound at a given concentration, and OD_control is the mean absorbance of the untreated positive control wells. Each compound was tested in three independent biological experiments performed in triplicate wells, and results are expressed as mean ± standard deviation (SD). The antibiofilm activity of QO-based QACs was compared to commercial reference compounds cetylpyridinium chloride (CPC), benzyldimethyldodecylammonium bromide (BAB), and the beta-lactam antibiotic cefotaxime. The crystal violet biofilm staining method was adapted from Odžak *et al.* [26].

### Time-kill kinetics assay

#### The time-kill kinetics

experiment was conducted with a representative Gram-positive bacterium, *Staphylococcus aureus* ATCC 25923. The overnight culture was diluted in fresh temperature-controlled MHB and shaken in an incubator at 37 ^∘^C and 220 rpm. Once the culture reached the exponential growth phase (OD_6__0__0_ ═ 0.3–0.5), it was diluted to a target cell concentration of 5 × 10^5^ CFU/mL. A total of 50 µL of the diluted culture was added to a microtiter plate containing the tested candidate compounds at concentrations of 1/2 MIC, MIC, and 2 × MIC. Prior to incubation at 37 ^∘^C, an aliquot of 10 µL was taken from each well and diluted in 990 µL MHB (1:100 dilution). The prepared dilution of the bacterial culture was subsequently serially diluted to achieve a final dilution factor of 10^6^. From each dilution, 20 µL was inoculated in duplicate onto solid Mueller-Hinton agar to determine the total number of viable cells at the start of the experiment by counting colonies formed after overnight incubation at 37 ^∘^C. The actual starting inoculum, determined by viable plate counting at *t* ═ 0, ranged from 4.75 × 10^6^ to 1.04 × 10^7^ CFU/mL across treatment groups. Sampling and plating were repeated at 30-minute intervals over a total period of 240 min to obtain time-kill curves illustrating the change in viable cell count with the duration of incubation of the bacteria with the antimicrobial compounds. The limit of detection (LOD) of the plating method was 2.5 × 10^4^ CFU/mL (log_1__0_ ═ 4.40), established by the lowest countable dilution. Samples yielding zero colonies were plotted at the LOD. All experiments were performed in duplicate, and results are presented as mean log_1__0_ CFU/mL ± standard deviation. To account for minor inter-treatment variation in the starting inoculum, time-kill curves were normalized to a common starting point.

### Cytotoxicity

The cytotoxicity of the newly synthesized QO-QACs was assessed using the CellTiter 96^®^ Aqueous One Solution Cell Proliferation Assay (Promega, Madison, WI, USA) according to the manufacturer’s guidelines. Human cell lines, specifically retinal pigment epithelial cells (RPE1) and human embryonic kidney cells (HEK293), were cultured at 37 ^∘^C in a humidified environment with 5% CO2. The compounds were dissolved in the appropriate culture medium and serially diluted in a microtiter plate within a concentration range of 250–0.24 µM. Subsequently, 5,000 cells per well were added, and the plates were incubated for 48 h under the same conditions. After incubation, 20 µL of MTS reagent was added to each well. Following three hours of incubation with the reagent, absorbance was measured at a wavelength of 490 nm using a Tecan Infinite 200 PRO plate reader. The results obtained were compared to those of commercial CPC and BAB, evaluated under identical conditions. The half-maximal inhibitory concentration (IC50), indicating the compound concentration at which 50% cell viability inhibition occurs, was determined via four-parameter logistic nonlinear regression using GraFit 6.0 software. Each compound was tested in three independent biological experiments, and results are expressed as mean IC50 ± SD. To evaluate the therapeutic selectivity of each compound, the selectivity index (SI) was calculated using the formula SI = IC50 (µM) / MIC (µM), with MIC values obtained in Mueller–Hinton broth against *Staphylococcus aureus*ATCC 25923 (SI1) and *Listeria monocytogenes*ATCC 7644 (SI2). SI values greater than 1 indicate a preference for antibacterial activity over mammalian cell toxicity.

### *In vivo* zebrafish embryotoxicity test

A zebrafish embryotoxicity test (ZET) was conducted following Organisation for Economic Co-operation and Development (OECD) 236 (2013) guidelines [27], as previously described in Crnčević et al. (2024) [28]. Embryos from three independent spawning events were randomly allocated to treatment groups. For dose range-finding experiments, embryos were exposed to serial dilutions across a broad concentration range in two replicates (10 embryos per replicate): QO-_14_ (273.0–4.3 µM) and QO-_16_ (242.0–3.8 µM). The samples were dissolved in dimethyl sulfoxide (DMSO), ensuring the final DMSO concentration in the test solutions did not exceed 0.1% [v/v; OECD 236 (2013)]. E3 medium served as the negative control. The assay plates were incubated at 27.5 ± 0.5 ^∘^C (Innova 42 incubator, New Brunswick). Mortality assessments were conducted at 120 h post-fertilization (hpf) using an Olympus CKX41 inverted microscope equipped with a Leica EC3 digital camera and LAS EZ 3.2.0 software. The validity of the assay was confirmed by control mortality remaining below 10%, as per OECD 236 guidelines [27].

### Determination of cmc

Conductivity measurements were performed as previously described [29]. All experiments were conducted at a controlled temperature of (25 ± 0.01) ^∘^C using an Orion conductivity meter (model 018001) equipped with platinum electrodes. The cell constant (0.10501 ± 0.00002 cm^−1^) was established through calibration with aqueous potassium chloride solutions within a concentration range of 0.001–0.05 mol dm^−3^. The conductivity cell was connected to a precision component analyzer (Wayne-Kerr, model 6430A). The resistance (R) of the test solutions was measured at frequencies of 500, 800, 1000, and 2000 Hz, exhibiting a linear relationship between resistance and reciprocal frequency. The experimental procedure began with the preparation of 10 cm^3^ of ultrapure water in a glass reaction cell. Any dissolved CO2 was removed by briefly bubbling high-purity nitrogen through the solution. The reaction cell was then sealed with a Teflon lid and placed in a Thermo-Haake Circulator DC10-V15/B for thermal equilibration. Once thermal equilibrium was achieved, the resistance of the solution at the four frequencies was recorded. Subsequently, a known volume of concentrated surfactant solution was introduced into the cell via a micropipette, and resistance measurements were repeated. Between these operations, the test solution was thoroughly homogenized using a Teflon-coated magnetic stir bar driven by a Cyclone immiscible stirrer (model 1-100, HMC Europe). From the obtained values, graphs depicting the relationship between electrical conductivity and compound concentration were constructed, and the cmc was calculated from the intersection of the two linear regions. The uncertainty of the cmc values was estimated through error propagation from the standard errors of the slope and intercept parameters of both linear fits, as the limited quantity of synthesized compounds precluded independent replicate measurements. Data analysis and linear regression were conducted using Microsoft Excel.

### Quantitative assessment of molecular descriptors

Consensus calculated log*P* (cLog*P*) values and topological polar surface area (TPSA) were calculated using the SwissADME online tool (http://www.swissadme.ch, accessed on 23 January 2025) [30]. Simplified Molecular Input Line Entry System (SMILES) representations of the compounds, generated using ChemDraw [31] and listed in Table 1, served as input for the calculations. Molecular structures and TPSA distributions were visualized using Jmol (http://jmol.sourceforge.net, accessed on 23 January 2025).

**Table 1:** List of compound abbreviations and their corresponding SMILES notations utilized in ADME calculations

**Compound**	**SMILES**
QO-C_12_	CCCCCCCCCCCC[N@+](C1)(CC2)CC[C@@H]2C1=O.[Br-]
QO-C_14_	CCCCCCCCCCCCCC[N@+](C1)(CC2)CC[C@@H]2C1=O.[Br-]
QO-C_16_	CCCCCCCCCCCCCCCC[N@+](C1)(CC2)CC[C@@H]2C1=O.[Br-]
BAB	C[N+](CC1=CC=CC=C1)(C)CCCCCCCCCCCC.[Br-]

**Abbreviations:** ADME, absorption, distribution, metabolism, and excretion; BAB, benzyldimethyldodecylammonium bromide; QO-C_12_, *N*-dodecyl-3-oxoquinuclidinium bromide; QO-C_14_, *N*-tetradecyl-3-oxoquinuclidinium bromide; QO-C_16_, *N*-hexadecyl-3-oxoquinuclidinium bromide; SMILES, simplified molecular-input line-entry system.

### Propidium iodide (PI) uptake assay

The membranolytic potential of the synthesized QO-QAC derivatives was evaluated by monitoring propidium iodide (PI) uptake as an indicator of membrane integrity in *Staphylococcus aureus* ATCC 25923. PI is a membrane-impermeant fluorescent nucleic acid stain that penetrates only cells with compromised membranes, intercalating into DNA and resulting in a measurable increase in fluorescence.

An overnight bacterial culture was established in MHB at 37 ^∘^C and 220 rpm. The following day, the culture was diluted 1:20 in fresh temperature-controlled MHB and incubated under the same conditions until the exponential growth phase was reached (OD600 = 0.3–0.5). Cells were harvested by centrifugation at 4,500 × *g* for 10 min at room temperature, and the bacterial pellet was resuspended in phosphate-buffered saline (PBS) to a final OD600 of 1.5. PI fluorescence was measured in black 96-well microtiter plates (Porvair). Each well received 100 µL of the bacterial suspension and 100 µL of the test compound pre-diluted in PBS containing 42 nM PI, yielding a final PI concentration of 21 nM per well. Compounds were tested at four concentrations: 4×MIC, 2×MIC, MIC, and 1/2×MIC. An untreated bacterial suspension incubated with PI served as the positive control (PC; intact membrane, minimal PI uptake anticipated), while a PI-in-PBS-only well without bacteria served as the negative control (NC; background fluorescence). PI fluorescence was recorded at 10-minute intervals for approximately 340 min at 37 ^∘^C in a Tecan Infinite 200 PRO fluorometer with excitation at 536 nm and emission at 617 nm. Background PI fluorescence (NC) was subtracted from each measurement, and data analysis was performed using Microsoft Excel. All measurements were conducted in three independent biological replicates; results are expressed as mean ± standard error of the mean (SEM) (*n*= 3).

### Ethical statement

Animal housing and spawning were conducted in aquarium units approved by the Croatian Ministry of Agriculture (HR-POK-023), in accordance with Directive 2010/63/EU [32]. All experiments in this study involved non-protected embryonal stages (up to 120 hpf), which do not require approval from animal welfare authorities.

### Statistical analysis

Statistical analysis was conducted using GraphPad Prism 8.0.2 software. Conductivity data and PI uptake curves were plotted using Microsoft Excel.

The primary endpoints of this study were antibacterial MIC values across the tested bacterial strains and growth media (Tables 2 and 3) and cytotoxicity selectivity indices. Secondary analyses, including growth kinetics parameters, AUC for PI uptake, endpoint fluorescence, and biofilm inhibition at individual concentrations, are exploratory and reported as descriptive statistics (mean ± SD or mean ± SEM, as specified). No correction for multiple testing across compounds was applied for secondary exploratory endpoints.

**Table 2:** Minimum inhibitory concentrations (MICs/µM) of newly synthesized quinuclidin-3-one-based QACs compared to commercial standards: BAB and CPC

**Species**	**Compound**
**Gram-positive**	**Strain Origin**	**QO-C_12_** **_Mr__= 374.4_**	**QO-C_14_** **_Mr = 402.5_**	**QO-C_16_** **_Mr = 430.5_**	**BAB** **_Mr = 384.4_**	**CPC** **_Mr ═ 340_**
*Staphylococcus aureus*	ATCC 25923	63	**8**	**8**	10	4
*Staphylococcus aureus*	Clinical/MRSA	125	16	**8**	25	8
*Staphylococcus aureus*	ATCC 33591	125	16	**8**	25	6
*Bacillus cereus*	ATCC 14579	>250	63	31	25	16
*Listeria monocytogenes*	ATCC 7644	125	16	**4**	10	8
*Enterococcus faecalis*	ATCC 29212	>250	63	63	15	8
**Gram-negative**						
*Escherichia coli*	ATCC 25922	>250	63	31	63	16
*Salmonella enterica*	food isolate	>250	63	63	50	63
*Pseudomonas aeruginosa*	ATCC 27853	>250	250	>250	>125	250

MICs expressed in micromolar (µM) represent modal values obtained from three or more independent experiments. **Abbreviations:** ATCC, American Type Culture Collection; BAB, benzyldimethyldodecylammonium bromide; CPC, cetylpyridinium chloride; MIC, minimum inhibitory concentration; MR, relative molecular mass; MRSA, methicillin-resistant *Staphylococcus aureus*; QAC, quaternary ammonium compound; QO-C_12_, *N*-dodecyl-3-oxoquinuclidinium bromide; QO-C_14_, *N*-tetradecyl-3-oxoquinuclidinium bromide; QO-C_16_, *N*-hexadecyl-3-oxoquinuclidinium bromide; µM, micromolar.

LC_5__0_ values for zebrafish embryotoxicity were determined through nonlinear regression using a four-parameter logistic model with variable slope in GraphPad Prism 8.0.2. Prior to fitting, mortality values underwent logarithmic transformation. Confidence intervals (95%) were calculated using the profile likelihood method, and model goodness-of-fit was evaluated using the coefficient of determination (R^2^) and residual analysis.

For the PI uptake assay, differences in area under the fluorescence–time curve and endpoint fluorescence (F340) across concentrations were analyzed using one-way analysis of variance (ANOVA) followed by Tukey’s Honestly Significant Difference (HSD) post-hoc test, applied separately for each compound. Statistical significance was accepted at *P* < 0.05. All fluorescence values are expressed as mean ± SEM of three independent biological replicates (*n* ═ 3).

Growth kinetics parameters (AUC, lag phase, maximum growth rate, and growth amplitude; Table 4) are reported as descriptive statistics (mean ± SD, *n* ═ 3); no inferential statistical comparisons were made between treatment groups for these parameters.

**Table 3:** Minimum inhibitory concentrations (MIC, µM) of QO-QACs and BAB against *Staphylococcus aureus* ATCC 25923, assessed in three different growth media

		**MIC (µM)**		**Fold change^b^**
**Compound**	**MHB**	**CA-MHB**	**DMEM**	**MHB/DMEM**
QO-C_12_	62.5	62.5	1.95	32×
QO-C_14_	7.81	7.81	1.95	4×
QO-C_16_	7.81	7.81	1.95	4×
BAB ^a^	10	—	—	—

^a^BAB refers to benzyldimethyldodecylammonium bromide, a commercial QAC utilized as a positive control. MHB stands for Mueller-Hinton broth; CA-MHB indicates cation-adjusted Mueller-Hinton broth; DMEM denotes Dulbecco’s Modified Eagle Medium. ^b^Values are presented as modal values derived from three independent experiments. The fold-change is calculated as MIC(MHB) ÷ MIC(DMEM), with an uncertainty of ±1 twofold dilution step applicable to all fold-change values. — ═ not determined. **Abbreviations:** ATCC, American Type Culture Collection; BAB, benzyldimethyldodecylammonium bromide; CA-MHB, cation-adjusted Mueller-Hinton broth; DMEM, Dulbecco’s Modified Eagle Medium; MHB, Mueller-Hinton broth; MIC, minimum inhibitory concentration; QAC, quaternary ammonium compound; QO-C_12_, *N*-dodecyl-3-oxoquinuclidinium bromide; QO-C_14_, *N*-tetradecyl-3-oxoquinuclidinium bromide; QO-C_16_, *N*-hexadecyl-3-oxoquinuclidinium bromide; QO-QACs, quinuclidin-3-one-based quaternary ammonium compounds.

Biofilm inhibition data are reported as mean ± SD from three independent biological experiments conducted in triplicate wells. Due to the limited number of tested concentrations (four), no dose-response curve fitting was performed; results are presented as percentage inhibition at each concentration.

## Results

### Antibacterial activity

Three QO-QACs with varying alkyl chain lengths (C_1__2_, C_1__4_, and C_16_) were synthesized with high yields through N-alkylation of quinuclidin-3-one using the corresponding alkyl bromides (Scheme 1).

**Scheme 1.**
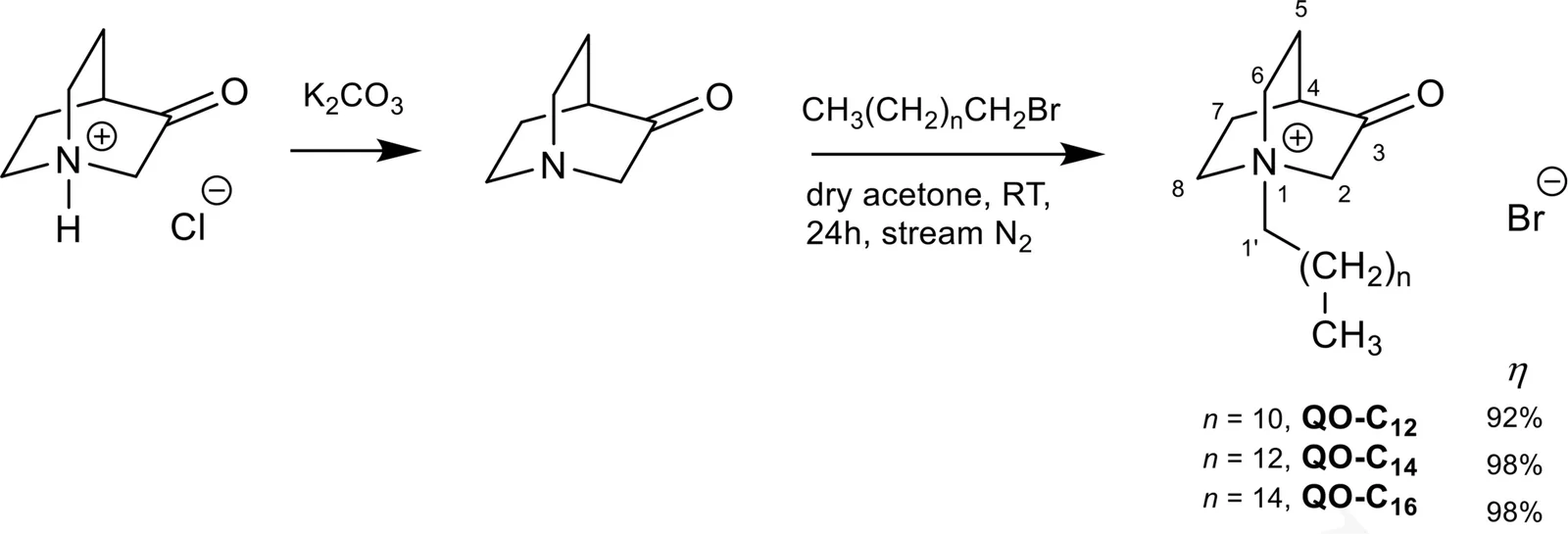
**Synthesis of the quinuclidin-3-one-based quaternary ammonium compounds QO-C_12_, QO-C_14_, and QO-C_16_.** Quinuclidin-3-one, obtained from its hydrochloride precursor, was N-alkylated with 1-bromododecane, 1-bromotetradecane, and 1-bromohexadecane in dry acetone at RT for 24 h under N2 to afford the corresponding C12, C14, and C16 derivatives in high isolated yields (92%, 98%, and 98%, respectively). **Abbreviations:** N2, nitrogen; QO-C_12_, *N*-dodecyl-3-oxoquinuclidinium bromide; QO-C14, *N*-tetradecyl-3-oxoquinuclidinium bromide; QO-C_16_, *N*-hexadecyl-3-oxoquinuclidinium bromide; RT, room temperature.

The effect of the C-3 substituent on the antibacterial properties of the QACs was evaluated by comparing MICs with those of two commercial QACs: BAB and CPC (Table 2). The MIC values indicate that QO-C_1__4_ and QO-C_16_ exhibited superior activity against all tested Gram-positive strains in comparison to their C_1__2_ analogue, with QO-C_16_ demonstrating the lowest MIC values—8 µM against *S. aureus* ATCC 25923, ATCC 33591, and MRSA, and 4 µM against *L. monocytogenes* ATCC 7644. These values were equal to or lower than those of the commercial standards BAB (10 µM) and CPC (4–8 µM) against the same strains. The C_1__4_ and C_16_ derivatives exhibited enhanced antibacterial activity compared to their C_1__2_ analogue. Specifically, QO-QACs with C_1__4_ and C_16_ chains demonstrated low MICs against *Staphylococcus aureus* ATCC 25923, *Staphylococcus aureus* ATCC 33591, and MRSA. These findings are consistent with literature reports indicating that QACs with longer alkyl chains generally display increased antibacterial efficacy [6]. Notably, QO-C_16_ exhibited the lowest single-digit MIC values against Gram-positive bacteria, including *Listeria monocytogenes* ATCC 7644 (MIC = 4 µM), suggesting the potential for an additional, species-specific mechanism of action.

Comparing the MIC values of QO-QACs against representative *S. aureus* strains revealed that the obtained MIC values were significantly lower than those previously reported for structurally related quinuclidine-based QACs. This underscores the superior antibacterial potency of the present compounds relative to earlier investigated analogues.

Previously synthesized QACs derived from 3-hydroxy- and 3-chloroquinuclidine, quaternized with benzyl or para-bromo, para-chloro, and para-nitro substituents, exhibited MIC values ranging from 300 to 17 µM [33]. Additionally, quinuclidin-3-ol-derived QACs demonstrated antibacterial activity with MICs ranging from 359 to 2 µM, while QACs based on 3-aminoquinuclidine showed MIC values from 100 to 25 µM [21, 22]. In contrast, the current quinuclidin-3-one QACs displayed lower MIC values, thereby highlighting their enhanced antibacterial profile and suggesting that the quinuclidin-3-one scaffold is a particularly promising framework for further structural optimization.

To further evaluate the antimicrobial potential of the synthesized compounds, we assessed their susceptibility to efflux-mediated resistance using CCCP, an ATP synthesis inhibitor [34]. As ATP is essential for the activity of efflux pumps, its depletion compromises their function, allowing us to determine whether the compounds are substrates for efflux pumps. *Staphylococcus aureus* ATCC 33591, a strain known to possess efflux pump (e.g., QacC) genes, was utilized in this assay. The MIC values of the tested QO-QACs remained unchanged in the presence of CCCP, indicating that these compounds are not efficiently recognized or expelled by efflux systems. This suggests low susceptibility to efflux-mediated resistance under the tested conditions [35]. However, it should be noted that the CCCP assay serves as an indirect measure and does not rule out other potential resistance mechanisms; more definitive experiments, such as serial passaging under sub-MIC exposure, represent a direction for future research.

Collectively, the results demonstrate that QO-C_1__4_ and QO-C_16_ exhibit potent antibacterial activity against clinically relevant Gram-positive pathogens, including methicillin-resistant *S. aureus* and *L. monocytogenes*, with MIC values comparable to or lower than those of commercial QAC standards (Table 2). The unchanged MIC values in the presence of CCCP indicate poor recognition of these compounds by ATP-dependent efflux transporters, suggesting low susceptibility to efflux-mediated resistance under the tested conditions. These characteristics collectively support the further development of quinuclidin-3-one-based QACs as next-generation antimicrobial agents.

### Antibacterial activity in different nutrient media

In previous research, we demonstrated that the type and composition of the growth medium significantly influence the antibacterial properties of QACs [26]. A major factor is the presence of cations in the growth medium, whose positive charge can enhance electrostatic interactions between the amphiphile and the negatively charged bacterial cell envelope, thereby facilitating initial adsorption to the cell surface. To evaluate the role of media composition on the antibacterial properties of selected compounds against the Gram-positive representative *Staphylococcus aureus* ATCC 25923 (Table 3), we examined the antibacterial properties in two media: CA-MHB and Dulbecco’s Modified Eagle Medium (DMEM).

The main component of MHB is casein, a negatively charged protein that can hinder the electrostatic interactions between QACs and the negatively charged components of the bacterial cell wall. Consequently, the presence of casein may reduce QAC efficacy by obstructing the initial binding step critical for their membranolytic action. In contrast, CA-MHB, which contains sodium and magnesium in the form of monovalent and divalent cations, is commonly used in antimicrobial susceptibility testing. These cations may help neutralize the inhibitory effects of casein and its degradation products, thereby enhancing electrostatic interactions between QACs and the bacterial membrane and potentially leading to lower observed MIC values.

Remarkably, the MIC values of the QO-QAC candidates in CA-MHB remained unchanged compared to those in standard MHB (Table 2): QO-C_1__4_ and QO-C_16_ exhibited equal potency (MIC = 7.81 µM), approximately 8-fold lower than QO-C_1__2_ (62.5 µM), and comparable to or slightly more active than the reference compound benzyldimethyldodecylammonium bromide (BAB; MIC = 10 µM). This observation indicates that the divalent cation supplementation (Ca^2+^ and Mg^2+^) characteristic of CA-MHB does not modulate the activity of these compounds, suggesting that their membrane interaction proceeds through a mechanism unaffected by physiological cation concentrations—contrasting with aminoglycosides and polymyxins, whose activity is sensitive to divalent cation levels.

To further assess the impact of nutrient composition on antibacterial activity, MIC values were determined in DMEM, a medium designed to support eukaryotic cell growth. Key components of DMEM, such as glucose, glutamine, pyruvate, lipoic acid, vitamin B_1__2_, biotin, and ascorbic acid, support diverse metabolic pathways relevant to bacterial physiology, while phenol red enables monitoring of metabolic changes during growth [36].

**Figure 1.**
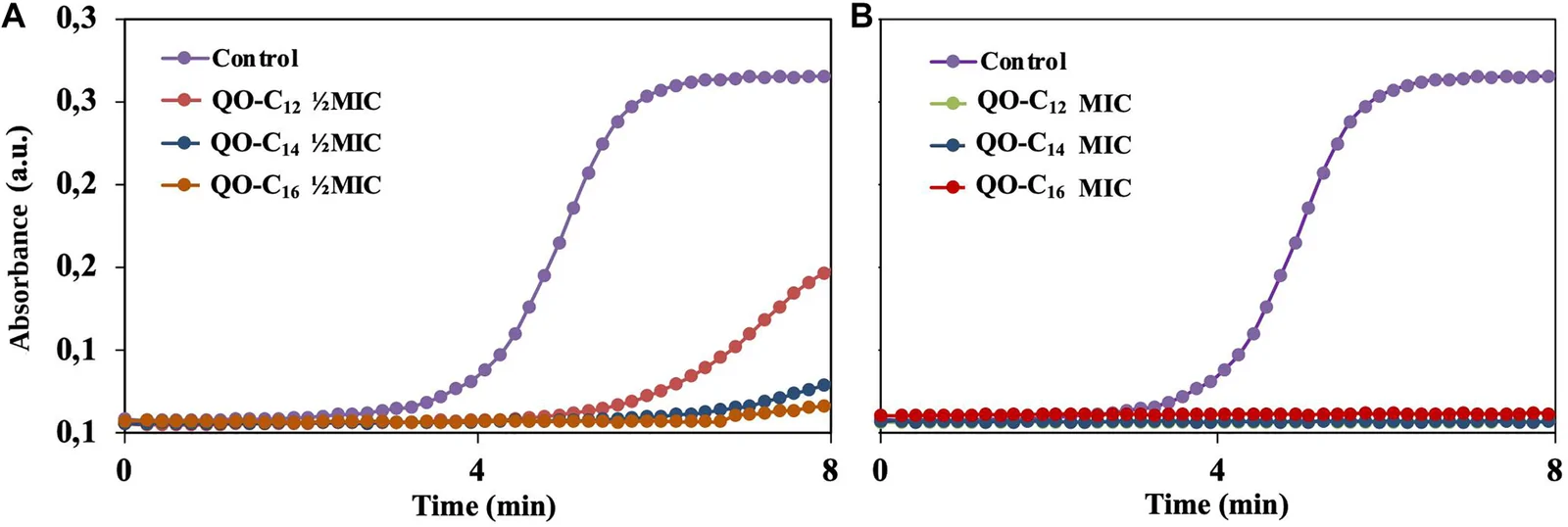
**Time-resolved growth curves of *Listeria monocytogenes* ATCC 7644 in the presence of quinuclidin-3-one quaternary ammonium compounds QO-C_12_, QO-C_14_, and QO-C_16_.** (A) Growth at 1/2 MIC concentrations; (B) Growth at MIC concentrations. Bacteria (5 × 10^4^ CFU/mL) were incubated in Mueller-Hinton broth at 37 ^∘^C with continuous shaking at 220 rpm. Optical density at 595 nm (OD_5__9__5_) was monitored at 10-minute intervals over approximately 11 h using a Tecan Infinite 200 PRO plate reader. Data are presented as mean ± SD (*n* ═ 3 independent biological replicates, each performed in triplicate). The untreated control exhibited typical sigmoidal growth with a lag phase of 182 ± 6 minutes. At 1/2 MIC, growth suppression was sustained for approximately 295 minutes with QO-C_12_, 392 ± 6 minutes with QO-C_14_, and 445 minutes with QO-C_16_, corresponding to growth inhibition (based on area under the curve) of 62.8%, 87.6%, and 91.6%, respectively. At MIC, all three compounds completely suppressed bacterial growth throughout the observation period, resulting in over 99% growth inhibition. Growth parameters, including AUC, lag phase, maximum growth rate, and amplitude, are summarized in the Growth Parameters sheet (Table 4). **Abbreviations:** ATCC, American Type Culture Collection; AUC, area under the curve; CFU/mL, colony-forming units per milliliter; MIC, minimum inhibitory concentration; OD595, optical density at 595 nm; QO-C_12_, N-dodecyl-3-oxoquinuclidinium bromide; QO-C_14_, *N*-tetradecyl-3-oxoquinuclidinium bromide; QO-C_16_, *N*-hexadecyl-3-oxoquinuclidinium bromide; rpm, revolutions per minute; SD, standard deviation.

Compared to the MIC values determined in CA-MHB and MHB, significantly lower MICs were observed in DMEM for all three candidate compounds (Table 3). In DMEM, all QO-ketones converged to the same MIC of 1.95 µM, corresponding to a 32-fold reduction for QO-C_1__2_ and a 4-fold reduction for QO-C_1__4_ and QO-C_16_ relative to broth-based media. This convergence is particularly noteworthy, as it eliminates the chain-length-dependent potency differences observed in MHB and CA-MHB. Importantly, bacterial incubation in DMEM was conducted under conditions identical to those used for MHB and CA-MHB (37 ^∘^C, ambient atmosphere), and active growth of *S. aureus* in unsupplemented DMEM control wells confirmed that the observed MIC reductions reflect genuine changes in compound potency rather than impaired bacterial growth. Furthermore, the DMEM used in this study contained no fetal bovine serum or other protein supplements, providing a protein-free environment that minimizes sequestration of the cationic amphiphiles.

The reason for this may be the absence of anionic peptides and amino acids in DMEM, which—unlike casein-derived peptides present in MHB—cannot sequester the cationic amphiphiles and reduce their free concentration available for membrane interaction.A synergistic contribution from DMEM components with documented antimicrobial properties (e.g., lipoic acid and ascorbic acid) cannot be excluded. The presence of nutrients may also influence the interaction between antimicrobial agents and bacteria. For instance, glucose and other bacterial metabolites could alter the composition of the bacterial cell wall and membrane, thereby affecting how QACs and other antimicrobial agents bind to the bacterial cell [34]. Comparative MIC determinations for BAB and CPC in CA-MHB and DMEM were not performed in the current study, as medium-dependent effects of classical long-chain QACs have been previously characterized [23, 37, 38]; such comparisons represent a direction for future research.

Based on these results, the composition of the culture medium appears to be a critical determinant of measured MICs, with both the type and concentration of its constituents potentially influencing the apparent antimicrobial efficacy as well as the rate and physiological state of bacterial growth.These findings also have practical implications: the MIC in DMEM may be more representative of activity under conditions of minimal protein binding (e.g., topical or intracellular applications), whereas MHB-derived values provide a more conservative estimate of potency under protein-rich physiological conditions.

### Influence of QO-based quaternary ammonium compounds on the growth dynamics of Listeria monocytogenes ATCC 7644

Investigation of bacterial growth kinetics provides critical insights into the growth dynamics of microorganisms in response to antimicrobial agents. Due to the potent antibacterial activity of QO-QACs against the Gram-positive bacterium *Listeria monocytogenes* ATCC 7644, a detailed analysis of growth kinetics was conducted to elucidate the effects of these compounds on bacterial proliferation (Figure 1).

**Table 4:** Growth kinetics parameters of *Listeria monocytogenes* ATCC 7644 in the presence of quinuclidin-3-one quaternary ammonium compounds (QO-QACs) at 1/2 MIC and MIC concentrations

**Treatment**	**AUC** **(OD·min)**	**Lag phase** **(min)**	**µ_max_** **(OD/h)**	**Amplitude** **(ΔOD)**	**Inhibition** **(%)**
Control	76.9 ± 0.1	182 ± 6	0.124 ± 0.001	0.209 ± 0.001	—
QO-C_12_ 1/2MIC	28.6 ± 0.1	295 ± 0	0.050 ± 0.001	0.147 ± 0.001	62.8
QO-C_14_ 1/2MIC	9.5 ± 0.1	392 ± 6	0.021 ± 0.001	0.055 ± 0.000	87.6
QO-C_16_ 1/2MIC	6.5 ± 0.0	445 ± 0	0.020 ± 0.002	0.054 ± 0.001	91.6
QO-C_12_ MIC	0.4 ± 0.1	NG	0.004 ± 0.002	0.002 ± 0.001	99.5
QO-C_14_ MIC	0.5 ± 0.1	NG	0.003 ± 0.002	0.002 ± 0.001	99.4
QO-C_16_ MIC	3.3 ± 0.1	NG	0.004 ± 0.003	0.002 ± 0.001	95.8

Values are expressed as mean ± SD (*n* ═ 3 independent biological replicates, each conducted in triplicate wells). AUC refers to the area under the growth curve, calculated using trapezoidal integration of background-corrected OD595 over approximately 655 minutes. µmax represents the maximum specific growth rate, determined from the steepest 30-minute sliding window. Amplitude is defined as the maximum background-corrected OD minus the baseline OD. Inhibition indicates the percentage reduction in AUC compared to the untreated control. NG denotes no detectable growth during the observation period. The lag phase is defined as the interval required to exceed baseline plus three times the SD of the first five time points, sustained for at least three consecutive readings. **Abbreviations:** ATCC, American Type Culture Collection; AUC, area under the curve; MIC, minimum inhibitory concentration; NG, no detectable growth; OD, optical density; OD595, optical density at 595 nm; QO-C_12_, *N*-dodecyl-3-oxoquinuclidinium bromide; QO-C_14_, *N*-tetradecyl-3-oxoquinuclidinium bromide; QO-C_16_, *N*-hexadecyl-3-oxoquinuclidinium bromide; QO-QACs, quinuclidin-3-one quaternary ammonium compounds; SD, standard deviation; ΔOD, change in optical density; µmax, maximum specific growth rate.

**Figure 2.**
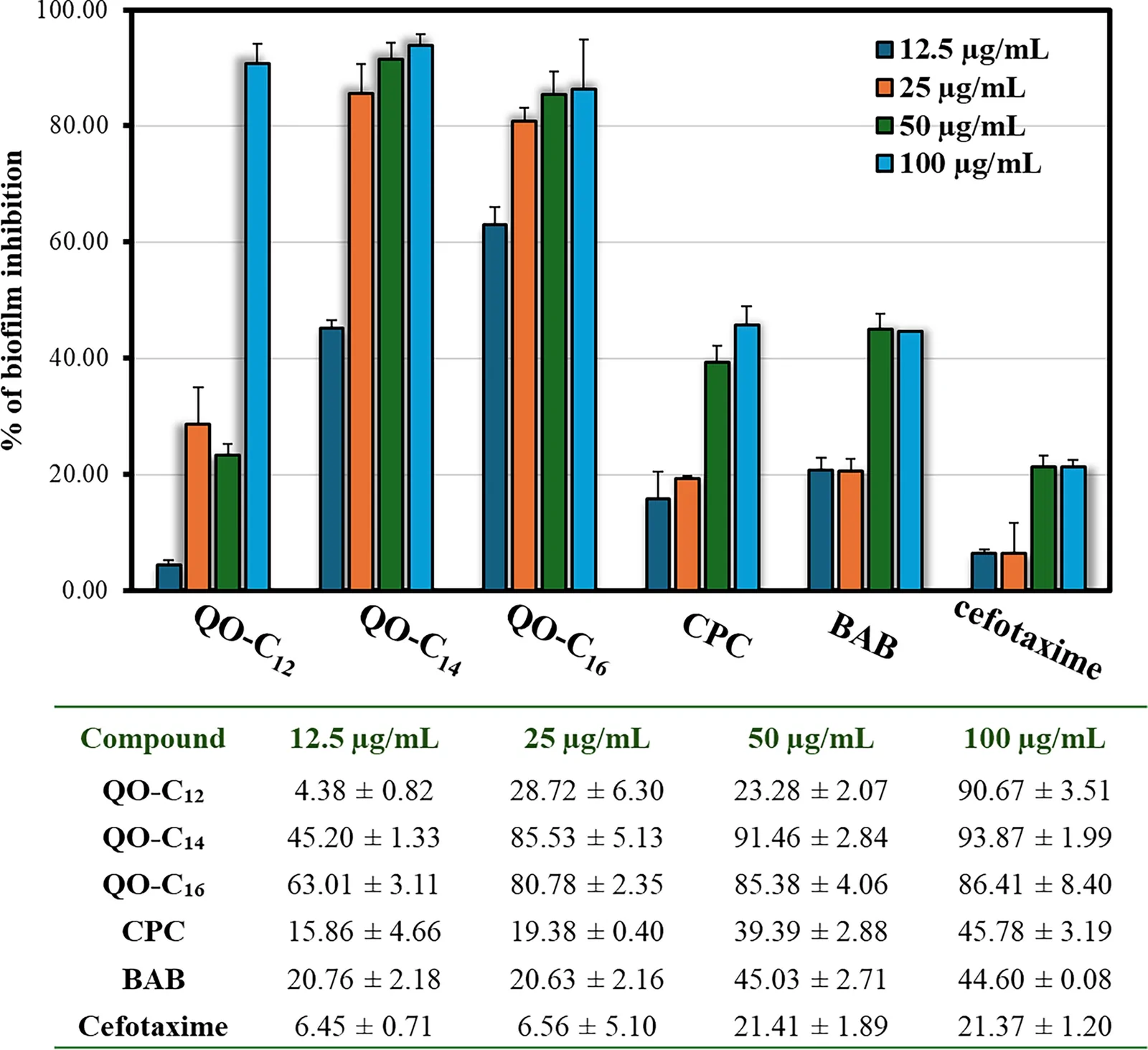
**Antibiofilm activity of QO-QACs against *Listeria monocytogenes* ATCC 7644, compared to commercial reference compounds CPC, BAB, and the beta-lactam antibiotic cefotaxime.** Biofilm inhibition was evaluated using the crystal violet staining method at four concentrations (12.5, 25, 50, and 100 µg/mL). Results are presented as percentage biofilm inhibition, calculated using the formula: % inhibitio*n* ═ 100 - (OD_treated_ / OD_control_) × 100, where OD_treated_ represents the absorbance of wells containing the test compound and OD_control_ denotes the mean absorbance of the untreated positive control. Bars represent the mean ± SD of three independent biological experiments conducted in triplicate wells (*n* ═ 3). **Abbreviations:** ATCC, American Type Culture Collection; BAB, benzyldimethyldodecylammonium bromide; CPC, cetylpyridinium chloride; OD, optical density; QO-QACs, quinuclidin-3-one-based quaternary ammonium compounds; SD, standard deviation.

**Figure 3.**
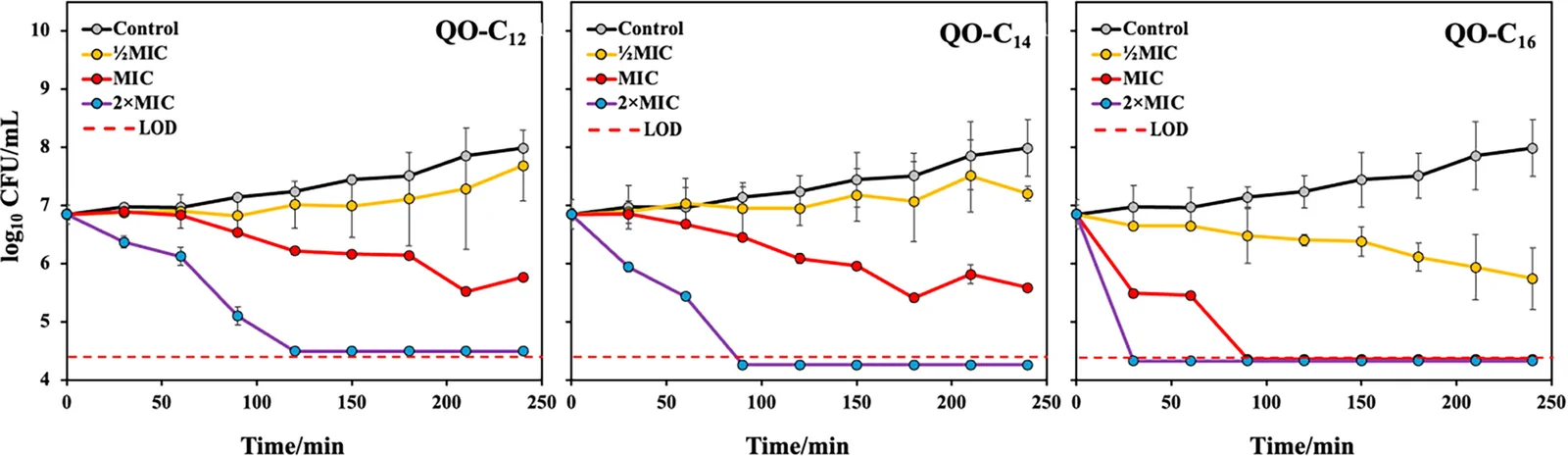
**Time-kill kinetics of quinuclidine-3-one quaternary ammonium compounds QO-C_12_, QO-C_14_, and QO-C_16_ against Staphylococcus aureus ATCC 25923 at 1/2MIC, MIC, and 2×MIC concentrations over a 4-hour incubation period.** Data are presented as mean log_10_ CFU/mL ± SD (*n* ═ 2 independent biological replicates with duplicate plating at each time point). The dashed red line indicates the limit of detection (LOD = 2.5 × 10^4^ CFU/mL; log_10_ ═ 4.40); samples yielding zero colonies are plotted at the LOD. The initial inoculum, determined by viable plate counting at t = 0, ranged from 4.75 × 10^6^ to 9.60 × 10^6^ CFU/mL across treatment groups. **Abbreviations:** ATCC, American Type Culture Collection; CFU/mL, colony-forming units per milliliter; LOD, limit of detection; MIC, minimum inhibitory concentration; QO-C_12_, N-dodecyl-3-oxoquinuclidinium bromide; QO-C_14_, N-tetradecyl-3-oxoquinuclidinium bromide; QO-C_16_, N-hexadecyl-3-oxoquinuclidinium bromide; SD, standard deviation.

**Figure 4.**
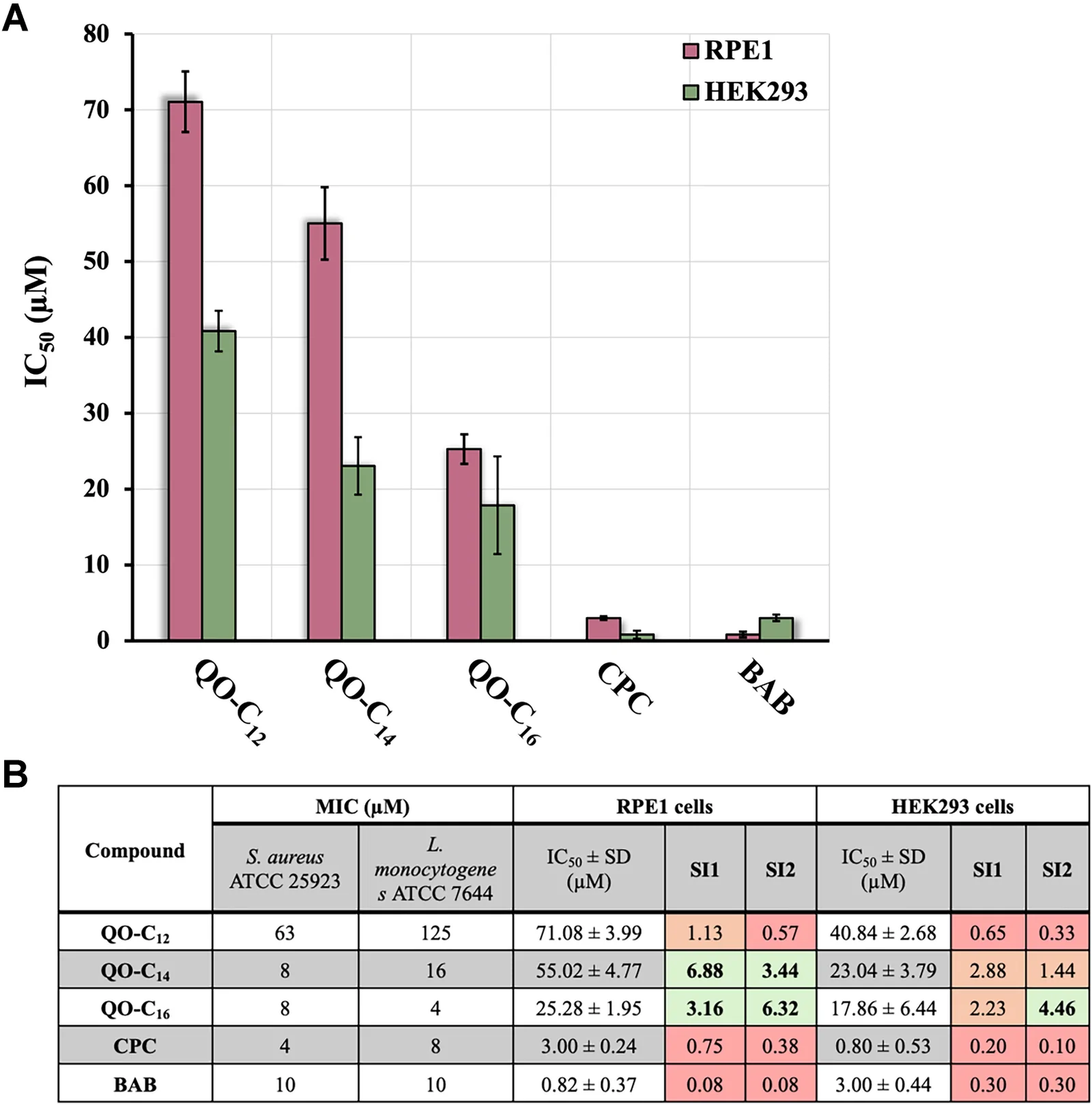
**Cytotoxicity and selectivity of QO-QACs against healthy human cell lines, compared to the commercial reference standards CPC and BAB.** (A) Half-maximal inhibitory concentrations (IC50, µM) were determined using four-parameter logistic nonlinear regression for RPE1 and HEK293 cells. Data are presented as mean ± SD (*n* = 3 independent biological experiments). (B) Minimum inhibitory concentrations (MIC, µM) and calculated selectivity indices, where SI1 indicates *Staphylococcus aureus* ATCC 25923 and SI2 indicates *Listeria monocytogenes* ATCC 7644 (MIC values assessed in Mueller-Hinton broth). Cell color coding in panel B: Green ≥ 3 (good selectivity); orange 1–3 (moderate selectivity); red < 1 (no selectivity). **Abbreviations:** ATCC, American Type Culture Collection; BAB, benzyldimethyldodecylammonium bromide; CPC, cetylpyridinium chloride; HEK293, human embryonic kidney 293 cells; IC50, half-maximal inhibitory concentration; MIC, minimum inhibitory concentration; QO-QACs, quinuclidin-3-one-based quaternary ammonium compounds; RPE1, retinal pigment epithelial cells; SD, standard deviation; SI, selectivity index; SI1, selectivity index calculated using Staphylococcus aureus ATCC 25923 MIC values; SI2, selectivity index calculated using Listeria monocytogenes ATCC 7644 MIC values.

Time-resolved growth analyses were conducted in comparison to an untreated control. The untreated control exhibited a typical bacterial growth curve, characterized by an initial *lag* phase followed by exponential growth, culminating in a plateau as the culture entered the stationary phase. The control’s *lag* phase averaged 182 ± 6 min (mean ± SD, *n* ═ 3), with a maximum growth rate of 0.124 ± 0.001 OD/h and a growth amplitude of 0.209 ± 0.001 ΔOD. In contrast, the growth curves obtained in the presence of antibacterial agents displayed a distinct pattern. At MIC levels, all three QO-QACs (QO-C12, QO-C14, and QO-C16) completely inhibited *L. monocytogenes*growth throughout the ∼11-hour observation period, with growth inhibition exceeding 99% as assessed by AUC analysis (Table 4).

At subinhibitory concentrations (1/2 MIC), growth inhibition was observed in a chain-length-dependent manner. QO-C12 extended the lag phase to approximately 295 min (compared to 182 ± 6 min for the control), with a reduced maximum growth rate of 0.050 ± 0.001 OD/h and a growth amplitude of 0.147 ± 0.001 ΔOD, corresponding to 62.8% growth inhibition. QO-C14 further extended the lag phase to 392 ± 6 min, with a maximum growth rate of 0.021 ± 0.001 OD/h and 87.6% growth inhibition. QO-C16 demonstrated the most prolonged suppression, with a lag phase of 445 min, a maximum growth rate of 0.020 ± 0.002 OD/h, and 91.6% growth inhibition (Table 4).

These results clearly indicate a chain-length-dependent effect on growth suppression, with QO-C16 identified as the most potent inhibitor, while all three compounds exhibited sustained growth-suppressive effects even below their MIC values.

### Biofilm inhibition assay

Bacterial biofilms represent complex and highly organized communities of bacteria embedded in a protective matrix, conferring increased resistance to antimicrobial agents. Biofilms are associated with numerous chronic infections and environmental challenges due to their tolerance to antimicrobial treatments. Understanding biofilm formation, structure, and resistance mechanisms is essential for developing effective strategies to prevent or treat biofilm-related infections [7, 39].

*Listeria monocytogenes* is a Gram-positive bacterium and a major causative agent of listeriosis, a foodborne illness that poses severe health risks, particularly for vulnerable populations such as neonates, the elderly, pregnant women, and immunocompromised individuals [40]. Biofilm formation significantly contributes to *L. monocytogenes* persistence and resistance to immune defense mechanisms and antibiotic treatments, complicating infection management [41, 42]. Cationic amphiphiles, such as QACs, are promising antibiofilm agents due to their positively charged nitrogen atom, which enhances electrostatic interactions with the negatively charged components of bacterial membranes and biofilm matrices, facilitating compound penetration and disruption of biofilm integrity [43].

In this study, the antibiofilm efficacy of QO-based QACs was evaluated against *L. monocytogenes* ATCC 7644 at four concentrations (12.5, 25, 50, and 100 µg/mL) and compared with commercial QACs, BAB and CPC, as well as the beta-lactam antibiotic cefotaxime (Figure 2).

QO-C14 and QO-C16 exhibited strong, concentration-dependent biofilm inhibition at all tested concentrations. QO-C14 inhibited *L. monocytogenes*biofilm formation by 93.87 ± 1.99% at 100 µg/mL, maintaining inhibition above 85% at concentrations as low as 25 µg/mL (85.53 ± 5.13%). QO-C16 achieved 86.41 ± 8.40% inhibition at 100 µg/mL and retained inhibition above 80% down to 25 µg/mL (80.78 ± 2.35%), with 63.01 ± 3.11% inhibition still observed at 12.5 µg/mL. These results indicate that both compounds maintain significant antibiofilm activity across the entire concentration range. QO-C12 also demonstrated potent inhibition at the highest tested concentration (90.67 ± 3.51% at 100 µg/mL), but inhibition decreased markedly at lower concentrations (23.28 ± 2.07% at 50 µg/mL, 28.72 ± 6.30% at 25 µg/mL, and 4.38 ± 0.82% at 12.5 µg/mL), indicating a narrow effective concentration range consistent with its shorter alkyl chain. In comparison, the commercial reference compounds exhibited substantially lower antibiofilm activity across all concentrations. CPC inhibited biofilm formation by 45.78 ± 3.19% and BAB by 44.60 ± 0.08% at 100 µg/mL, with both compounds demonstrating reduced efficacy at lower concentrations (CPC 15.86 ± 4.66% at 12.5 µg/mL; BAB 20.76 ± 2.18% at 12.5 µg/mL). Cefotaxime, a cell wall-targeting antibiotic lacking membrane-disruptive properties, displayed minimal antibiofilm activity across all concentrations (21.37 ± 1.20% at 100 µg/mL; 6.45 ± 0.71% at 12.5 µg/mL), consistent with the established tolerance of biofilms to conventional antibiotics.

Overall, QO-C14 and QO-C16 were identified as the most effective antibiofilm candidates, outperforming both commercial QAC standards and cefotaxime across all tested concentrations. Given the critical role of *L. monocytogenes*biofilms in persistent food-related and clinical infections, the potent antibiofilm activity of these compounds at concentrations as low as 12.5 µg/mL underscores their potential for practical applications in disinfection and therapeutic contexts.

### Time-kill kinetics assay

A commonly employed strategy for elucidating the mode of action of antimicrobial compounds involves monitoring bacterial viability over a defined incubation period. Compounds demonstrating significant antimicrobial activity can be classified as either bactericidal or bacteriostatic. Bactericidal agents are defined by their ability to eliminate ≥99.99% of the bacterial population within 24 h of exposure, while bacteriostatic agents inhibit bacterial growth without inducing cell death [44]. Experimental data are typically represented as the relationship between bacterial cell count (Log10 CFU/mL) and incubation time. A reduction of ≥3 Log10 CFU/mL in cell count is indicative of a bactericidal effect [44]. Figure 3 illustrates the kinetic profiles of candidate compounds’ effects on *Staphylococcus aureus* ATCC 25923 during a 4-hour incubation at 1/2 MIC, MIC, and 2 × MIC concentrations, providing insights into their mechanisms of action.

Analysis of the kinetic profiles of QO-C14 and QO-C16 reveals that QO-C16 exhibits superior antimicrobial activity across the evaluated time intervals. Specifically, QO-C16 eradicated the *Staphylococcus aureus* ATCC 25923 population within 2 h at its MIC. Furthermore, at 2 × MIC, QO-C16 demonstrated a rapid bactericidal effect, eliminating the bacterial population within just 30 min. In contrast, QO-C14 exhibited slower bactericidal kinetics, requiring prolonged exposure to achieve a comparable level of bacterial eradication. Time-kill kinetic analysis confirms that QO-C16 outperforms QO-C14, achieving rapid and sustained eradication of *S. aureus* at both MIC and 2 × MIC concentrations.

### Cytotoxicity

The toxicity of QACs represents a significant limitation, constraining their safe use and applicability in various settings and formulations. This concern has spurred research into QAC derivatives and alternative formulations with improved biocompatibility and reduced ecotoxicity, including biodegradable (“soft”) structures designed to limit environmental persistence and exposure of terrestrial and aquatic organisms [14]. Given the widespread use of QACs as disinfectants and antiseptics, minimizing cytotoxicity while maintaining antimicrobial efficacy is essential for safe human-relevant applications.

In this study, we evaluated the cytotoxicity of newly synthesized QO-C12, QO-C14, and QO-C16 against two healthy human cell lines, RPE1 and HEK293, and compared them with commercial QACs, CPC and BAB.

All three QO-QACs exhibited lower cytotoxicity than commercial standards across both cell lines (Figure 4). Cytotoxicity increased with alkyl chain length in both cell lines, consistent with the enhanced membrane affinity of longer-chain amphiphiles. The observed chain-length-dependent increase in cytotoxicity aligns with the calculated cLogP values, which progressively increase within the QO-QAC series (QO-C_1__2_: 1.65, QO-C_1__4_: 2.29, QO-C_16_: 2.95), reflecting enhanced membrane partitioning of more hydrophobic compounds and their greater propensity to interact with mammalian cell membranes. In RPE1 cells, the IC50 values were 71.08 ± 3.99 µM for QO-C12, 55.02 ± 4.77 µM for QO-C14, and 25.28 ± 1.95 µM for QO-C16. A similar trend was observed in HEK293 cells, with IC50 values of 40.84 ± 2.68 µM, 23.04 ± 3.79 µM, and 17.86 ± 6.44 µM for QO-C12, QO-C14, and QO-C16, respectively. IC50 values were consistently lower in HEK293 than in RPE1 cells for all tested compounds. This difference is attributed to the embryonic origin of HEK293 cells, which renders them inherently more sensitive to cytotoxic agents compared to differentiated somatic cells such as RPE1. Conversely, the commercial standards, CPC and BAB, exhibited high toxicity across both cell types, with IC50 values in the low micromolar to sub-micromolar range (CPC: RPE1 3.00 ± 0.24 µM, HEK293 0.80 ± 0.53 µM; BAB: RPE1 0.82 ± 0.37 µM, HEK293 3.00 ± 0.44 µM), confirming their well-documented toxicity toward mammalian cells (Figure 4).

In contrast, the quinuclidin-3-one derivatives demonstrated significantly lower toxicity toward both tested cell lines. The selectivity of the QO-QACs relative to their antibacterial efficacy was further assessed by calculating the selectivity index (SI = IC50 / MIC) for each compound (Figure 4). QO-C14 exhibited the most favorable selectivity against *S. aureus*, with SI1 values of 6.88 (RPE1) and 2.88 (HEK293). QO-C16 displayed the highest selectivity against *L. monocytogenes*, with SI2 values of 6.32 (RPE1) and 4.46 (HEK293), consistent with its exceptionally low MIC of 4 µM against this pathogen. In contrast, CPC and BAB demonstrated SI values below 1 in most conditions, indicating no meaningful selectivity advantage over mammalian cells. These findings confirm that the newly synthesized QO-QAC compounds represent promising antibacterial candidates with an improved safety profile compared to commercial standards, with QO-C14 and QO-C16 exhibiting the most favorable balance between antibacterial efficacy and mammalian cell selectivity.

### *In vivo* zebrafish embryotoxicity test

Embryotoxicity was observed in a concentration-dependent manner by exposing zebrafish *Danio rerio* embryos to two tested compounds, QO-C14 and QO-C16. The half-maximal lethal concentrations (LC50) for both compounds were derived from concentration-response curves, as illustrated in Figure 5. Among the tested compounds, QO-C16 exhibited greater toxicity, as indicated by a lower LC50 value (6.776 µM; 95% CI: 6.47–6.97) compared to QO-C14 (LC50 = 14.200 µM; 95% CI: 13.97–14.43). Nonlinear regression analysis revealed an excellent fit to the experimental data (R^2^ ═ 0.9957 for QO-C14 and R^2^ ═ 0.9937 for QO-C16).

**Figure 5.**
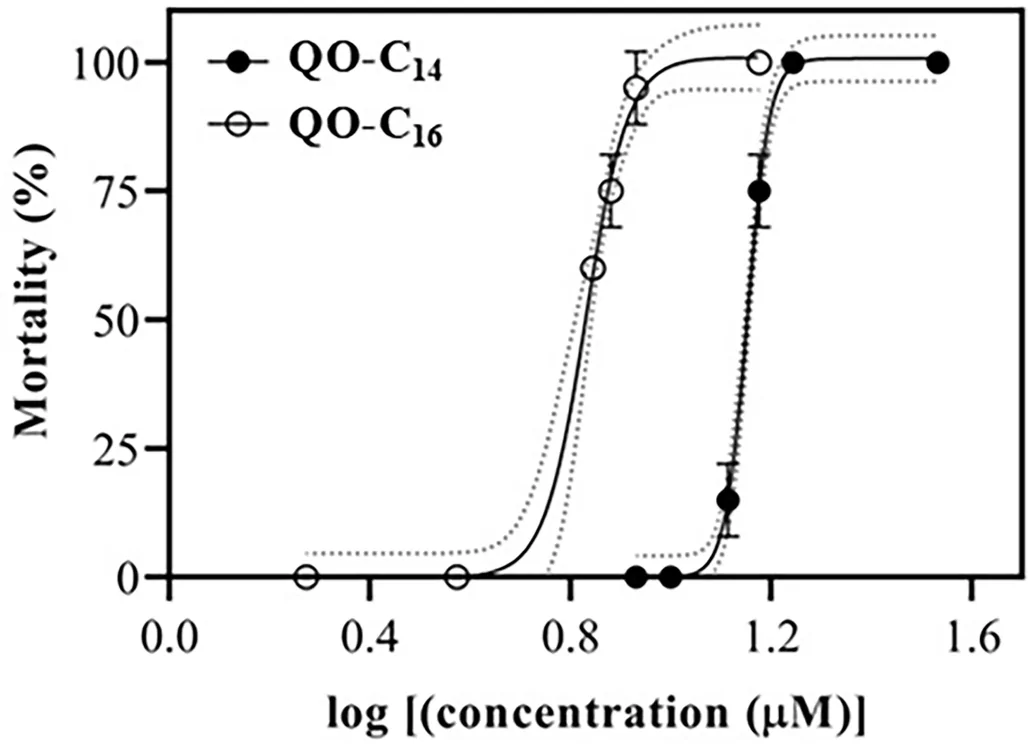
**Concentration-response curves utilized for calculating the mortality of Danio rerio following 120 h of exposure to QO-C_14_ and QO-C_16_.** Error bars indicate SD, while the dotted lines represent 95% CIs. **Abbreviations:** CI, confidence interval; QO-C_14_, *N*-tetradecyl-3-oxoquinuclidinium bromide; QO-C_16_, *N*-hexadecyl-3-oxoquinuclidinium bromide; SD, standard deviation.

**Figure 6.**
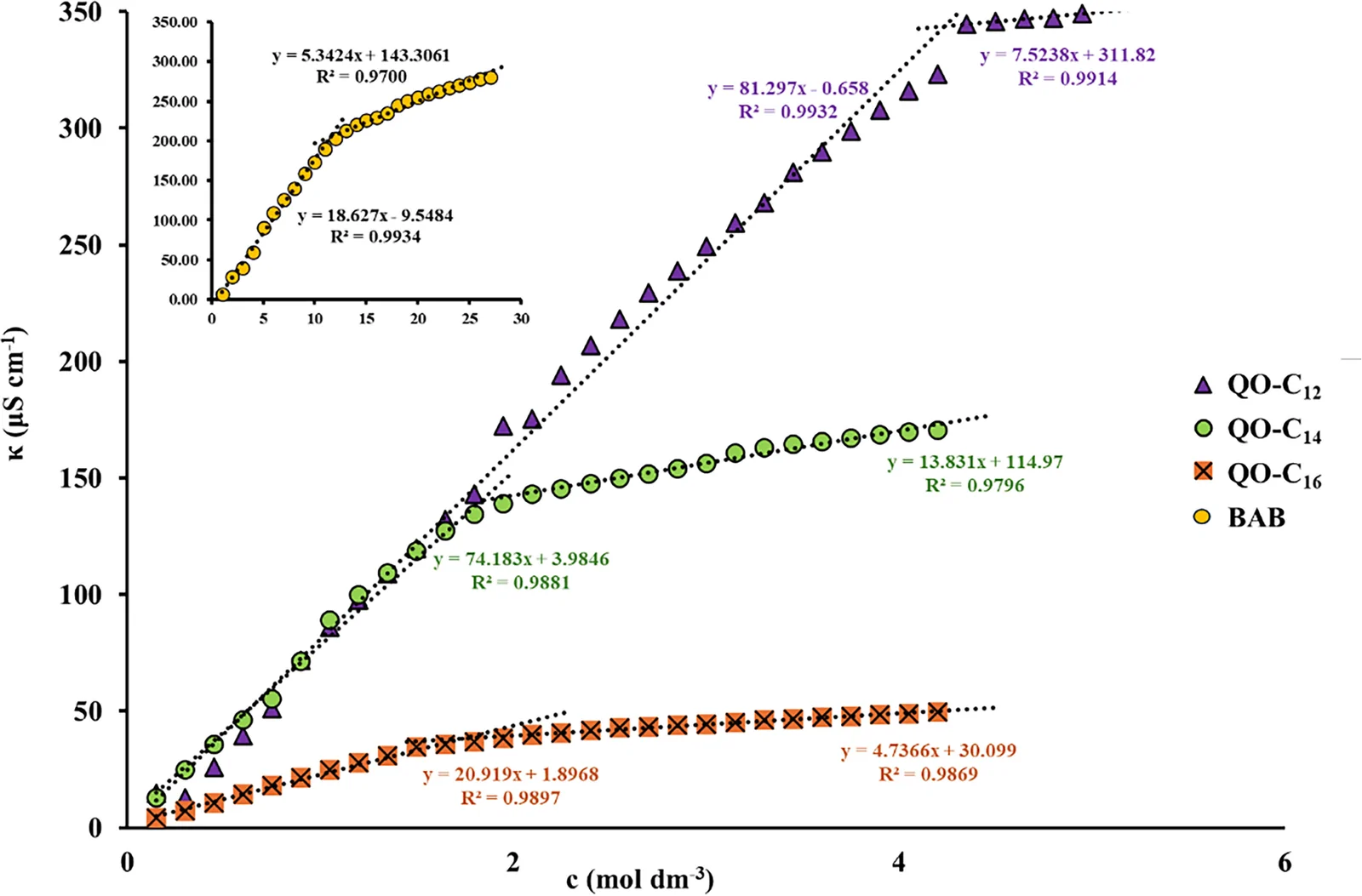
**Conductivity (κ) as a function of concentration (c) for quinuclidin-3-one-based quaternary ammonium compounds in aqueous solution at 25 ^∘^C.** The main panel shows the conductometric profiles of QO-C_12_, QO-C_14_, and QO-C_16_, whereas the inset shows the corresponding profile of the commercial reference compound BAB over a wider concentration range. Dotted lines represent linear regression fits to the pre-micellar and post-micellar regions, and their intersections were used to determine the critical micelle concentration (cmc). All tested compounds displayed characteristic surfactant behavior, with cmc decreasing as alkyl-chain length increased, consistent with enhanced hydrophobicity and more favorable micelle formation for the longer-chain derivatives. For the QO-QAC series, the cmc values were 4.24 ± 0.16 mM for QO-C_12_, 1.83 ± 0.17 mM for QO-C_14_, and 1.74 ± 0.16 mM for QO-C_16_. Notably, the steeper post-cmc slope observed for the shorter-chain derivatives, particularly QO-C_12_, is consistent with greater mobility of smaller aggregates in solution. **Abbreviations:** BAB, benzyldimethyldodecylammonium bromide; c, concentration; cmc, critical micelle concentration; QACs, quaternary ammonium compounds; QO-C_12_, *N*-dodecyl-3-oxoquinuclidinium bromide; QO-C_14_, *N*-tetradecyl-3-oxoquinuclidinium bromide; QO-C_16_, N-hexadecyl-3-oxoquinuclidinium bromide; κ, conductivity.

For comparison, the commercially available quaternary ammonium compound CPC demonstrated an LC50 value of 3.2 µM, indicating higher toxicity than both QO derivatives [28]. The lethality observed in the negative control group was below 5%.

### cmc

Conductometry is a highly sensitive and precise technique employed to examine interionic interactions, making it well-suited for investigating structural transformations in electrolyte solutions, such as ion-pairing and complexation [45–47]. The conductivity (*κ*) as a function of concentration (c) for the synthesized QACs is presented in Figure 6. The *cmc* is determined at the intersection of two distinct linear regions, each characterized by different slopes [48]. The *cmc* values, determined from the intersection of two linear regression lines fitted to the pre- and post-micellar conductivity regions, are reported with their estimated uncertainties as follows: QO-C_1__2_: 4.24 ± 0.16 mM, QO-C_1__4_: 1.83 ± 0.17 mM, and QO-C_16_: 1.74 ± 0.16 mM (Table 5). The reported uncertainties reflect the propagation of regression standard errors and do not represent standard deviations from independent replicate measurements. The *cmc* values for QO-based QACs align with previously reported findings for quinuclidin-3-ol, reinforcing the robustness of these results within the context of surfactant behavior [49]. The results indicate that all tested compounds exhibit characteristic surfactant properties, with *cmc* values corresponding to alkyl chain length. This observation is consistent with established surfactant behavior, wherein longer alkyl chains typically yield lower *cmc* values due to increased hydrophobicity and enhanced aggregation in solution, corroborating our prior research [11, 49]. Notably, there is a significant difference in the slope of the conductivity curve above the *cmc* for compounds with shorter hydrocarbon chains, as depicted in Figure 6. The steeper slope for shorter-chain QACs suggests faster migration of smaller aggregates within the solution. This phenomenon may be attributed to the smaller size and potentially lower viscosity of the micelles formed by these surfactants, facilitating a more rapid increase in conductivity as the concentration surpasses the *cmc*. These findings align with those reported by Quagliotto et al. and Zhao et al. [50], [47], further supporting the hypothesis that shorter alkyl chain surfactants form more compact aggregates with enhanced mobility in aqueous solutions. This behavior may have implications for the design and application of QACs, particularly in formulations requiring rapid response or improved conductivity. Additionally, the relationship between alkyl chain length and surfactant aggregation can be leveraged to optimize the performance of QACs across various industrial and environmental applications.

**Table 5:** Critical micelle concentration values of quinuclidine-derived QACs and their reference counterparts, accompanied by corresponding Ball-and-Stick molecular models

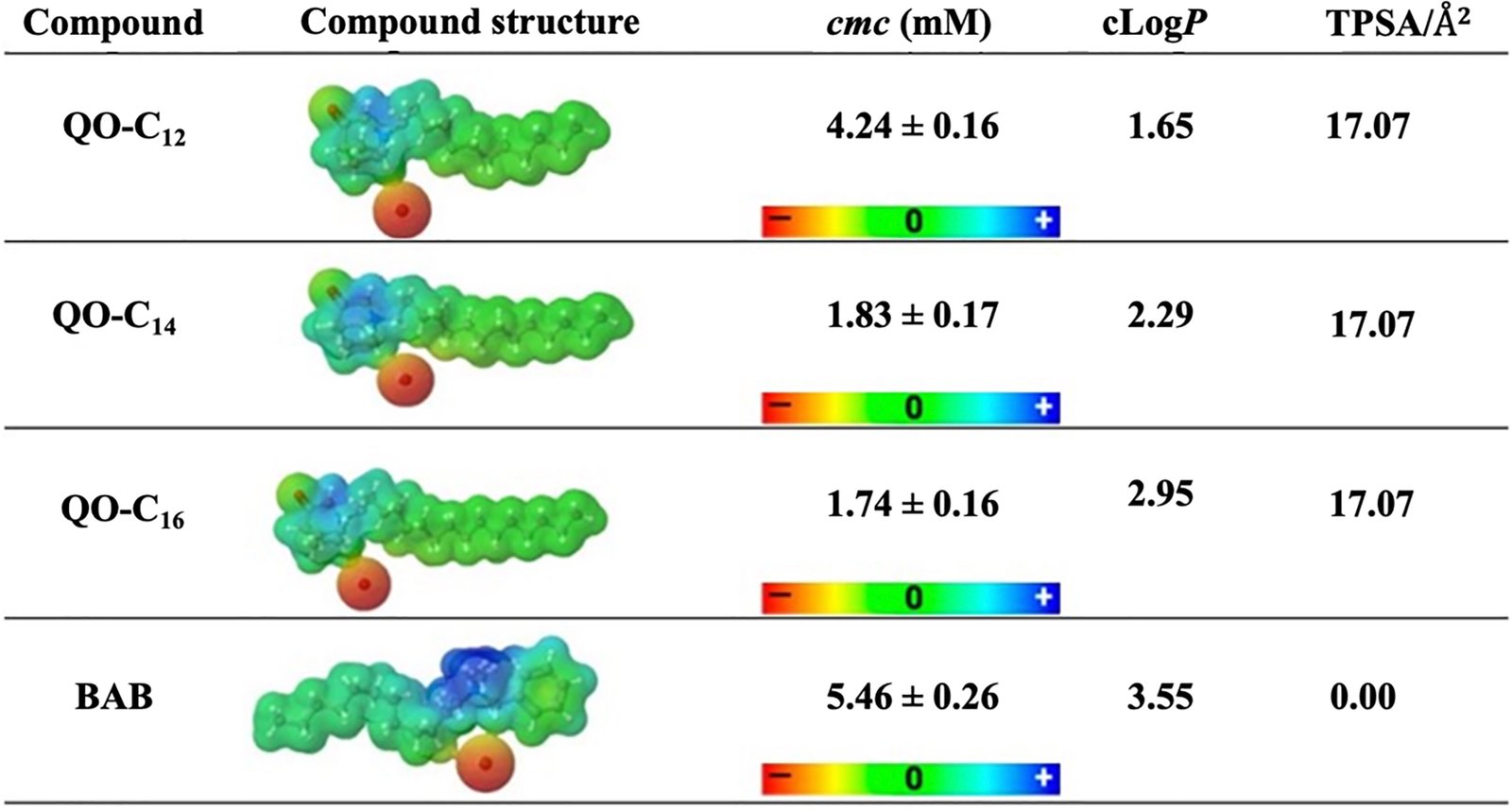

The table presents calculated Consensus Partition Coefficients (cLogP) and Topological Polar Surface Area (TPSA, Å^2^), offering essential insights into the amphiphilic balance and molecular polarity that are pertinent to their physicochemical and biological behavior. **Abbreviations:** BAB, benzyldimethyldodecylammonium bromide; cLogP, consensus partition coefficient; cmc, critical micelle concentration; QACs, quaternary ammonium compounds; QO-C_12_, *N*-dodecyl-3-oxoquinuclidinium bromide; QO-C_14_, *N*-tetradecyl-3-oxoquinuclidinium bromide; QO-C_16_, *N*-hexadecyl-3-oxoquinuclidinium bromide; TPSA, topological polar surface area; Å^2^, square angstroms.

### Lipophilicity (cLogP)

In this study, hydrophobicity predictions for all compounds were conducted using the SwissADME online tool, which computes an average cLog*P* value based on multiple estimations, including iLog*P*, xLog*P*, wLog*P*, and mLog*P* [30]. A higher cLog*P* value indicates enhanced partitioning into the lipid phase, serving as a valuable predictor of a compound’s distribution and behavior in complex biological environments.

The calculated cLog*P* values, presented in Table 5 reveal that quinuclidin-3-one QACs exhibit lower cLog*P* values than BAB, suggesting a more balanced distribution between lipid (hydrophobic) and aqueous (hydrophilic) phases. Compounds with lower cLog*P* values tend to be less hydrophobic, resulting in a greater affinity for the aqueous phase compared to the lipid phase. Consequently, these compounds are likely to exhibit limited membrane permeability, as their reduced hydrophobicity diminishes interaction with the lipid bilayer. Therefore, low cLog*P* compounds may demonstrate lower membrane penetration but enhanced solubility in aqueous environments, potentially influencing pharmacokinetic properties such as absorption [51].

Conversely, commercial BAB displays higher cLog*P* values, indicating increased hydrophobicity. This enhanced hydrophobic character suggests a greater tendency for this compound to partition into lipid-rich environments, such as cellular membranes. Additionally, increased hydrophobicity could facilitate interactions with hydrophobic sites in proteins, enzymes, or receptors, potentially contributing to their pharmacological activity [52].

### Mode of action

The membrane-disruptive potential of the synthesized QO-QAC derivatives was assessed using the PI uptake assay, as described in the Materials and Methods section.

**Figure 7.**
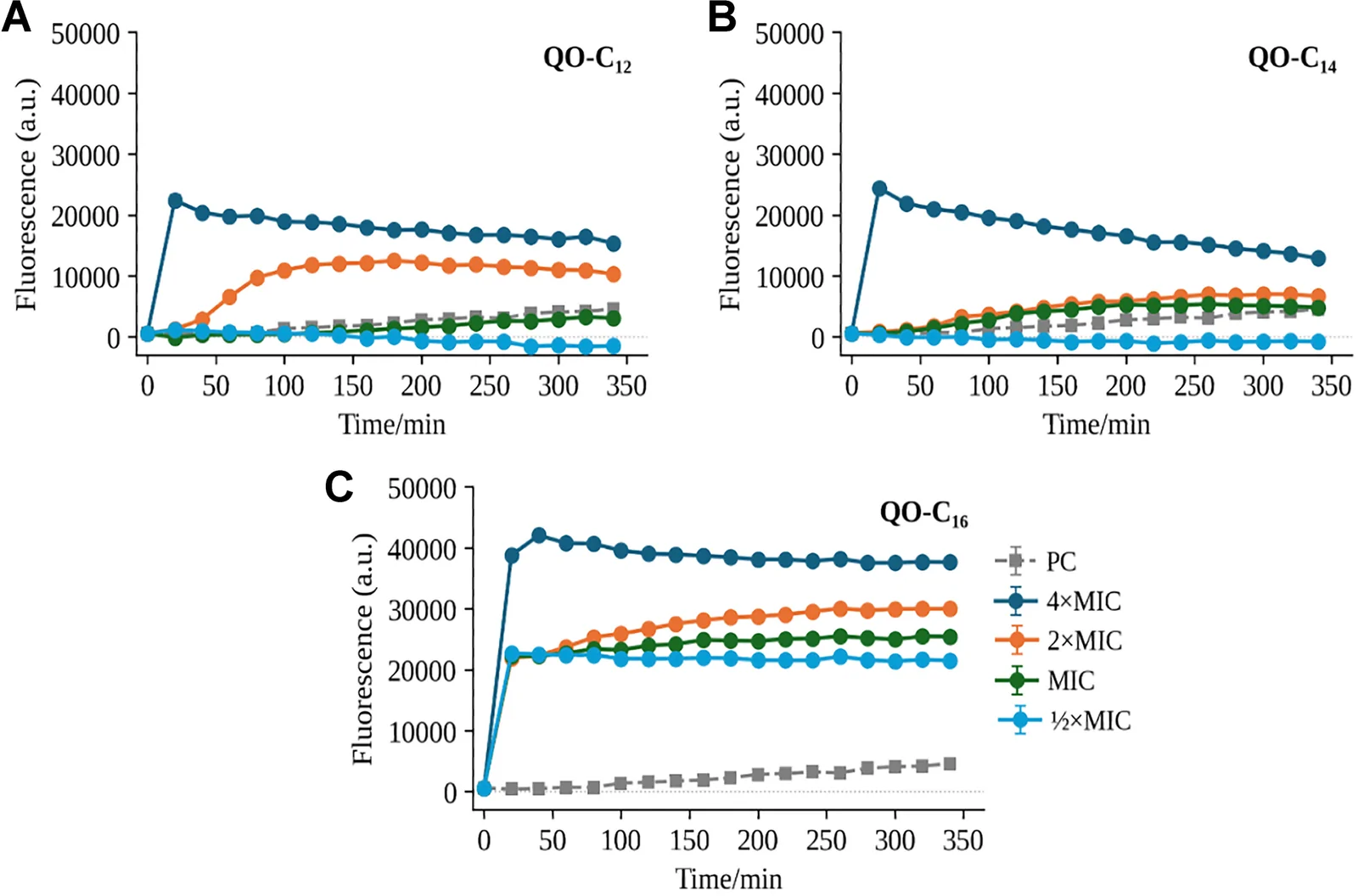
**Kinetics of PI uptake in** ***Staphylococcus aureus* ATCC 25923 upon exposure to quinuclidin-3-one quaternary ammonium compounds QO-C_12_, QO-C_14_, and QO-C_16_ at four concentrations (4×MIC, 2×MIC, MIC, and 1/2×MIC) over 340 minutes.** Bacterial suspensions (OD_600_ ═ 1.5 in PBS) were incubated with the test compounds in the presence of 21 nM PI at 37 ^∘^C, with fluorescence monitored at 10-minute intervals (excitation at 536 nm, emission at 617 nm). Background fluorescence (negative control: PI in PBS without bacteria) was subtracted. The positive control (PC) consisted of untreated bacterial cells incubated with PI. Data: Mean ± SEM (*n* ═ 3 independent biological replicates). Note: Y-axis scales differ between panels. Statistical comparisons by one-way ANOVA with Tukey’s HSD; *P < 0.05, **P < 0.01, ***P < 0.001. **Abbreviations:** ANOVA, analysis of variance; ATCC, American Type Culture Collection; HSD, honestly significant difference; MIC, minimum inhibitory concentration; nM, nanomolar; OD600, optical density at 600 nm; PBS, phosphate-buffered saline; PC, positive control; PI, propidium iodide; QO-C12, *N*-dodecyl-3-oxoquinuclidinium bromide; QO-C14, *N*-tetradecyl-3-oxoquinuclidinium bromide; QO-C16, *N*-hexadecyl-3-oxoquinuclidinium bromide; SEM, standard error of the mean.

The results presented in Figure 7 indicate that exposure to all four tested concentrations of QO-QACs resulted in a concentration-dependent, time-dependent increase in PI uptake, consistent with compromised bacterial membrane integrity. This was evidenced by a marked increase in fluorescence intensity compared to the untreated control, which exhibited negligible PI uptake, indicating sustained membrane integrity over the 340-minute observation period. At 4×MIC, a rapid and significant increase in PI fluorescence was observed for all three compounds within the first 20–40 min, indicating near-immediate membrane permeabilization. At the endpoint (*t* ═ 340 min), background-corrected fluorescence reached 15,354 ± 327 a.u. for QO-C12, 12,871 ± 334 a.u. for QO-C14, and 37,638 ± 349 a.u. for QO-C16. The substantially higher fluorescence intensity for QO-C16 across all concentrations reflects its greater membrane-disruptive potency, consistent with the enhanced hydrophobicity of its longer alkyl chain. At 2×MIC, the kinetic profiles displayed a more gradual increase, reaching endpoint values of 10,293 ± 601 a.u. (QO-C12), 6,654 ± 158 a.u. (QO-C14), and 29,997 ± 405 a.u. (QO-C16). At MIC, PI uptake remained measurable and time-dependent for all compounds, with endpoint fluorescence of 3,074 ± 129 a.u. (QO-C12), 4,794 ± 85 a.u. (QO-C14), and 25,422 ± 204 a.u. (QO-C16). Notably, at 1/2×MIC, QO-C12 and QO-C14 did not produce significant PI uptake above background (-1,513 ± 372 a.u. and -802 ± 320 a.u., respectively), indicating that sub-inhibitory concentrations of these compounds do not cause appreciable membrane disruption. In contrast, QO-C16 at 1/2×MIC elicited substantial PI uptake (21,494 ± 91 a.u. at *t* ═ 340 min), comparable to or exceeding the 4×MIC response of QO-C12 and QO-C14. This finding suggests that QO-C16 exerts significant membrane-permeabilizing activity even below its MIC, possibly reflecting an optimized amphiphilic balance that facilitates insertion into the lipid bilayer at lower compound concentrations. It is important to note that the observed differences in PI uptake at MIC and above-MIC treatments may be influenced by variations in bacterial cell density relative to MIC determination conditions. Specifically, a bacterial suspension of OD600 = 1.5 (approximately 10^9^ CFU/mL) was employed in the PI uptake experiments, compared with 5 × 10^5^ CFU/mL used for MIC determination. This adjustment was necessitated by methodological requirements related to fluorescence signal detection. Consequently, this variation in experimental conditions should be considered when interpreting the results.

Overall, these findings underscore the concentration-dependent efficacy of the QAC derivatives in disrupting the membranes of *S. aureus* ATCC 25923, highlighting their potential as membrane-active antimicrobial agents. While PI uptake provides clear evidence for membrane disruption, this assay alone does not elucidate the specific molecular targets or the precise nature of the interaction with the bacterial envelope. QACs are generally understood to disrupt the lipid bilayer through electrostatic interactions with negatively charged membrane components; however, secondary effects, such as perturbation of membrane proteins or induction of oxidative stress, cannot be excluded [50, 51]. Additional complementary approaches, such as leakage assays of intracellular components, electron microscopy to visualize membrane damage, or experiments using model membrane systems, would be valuable to determine whether the mechanism is purely lytic or involves other cellular processes [52].

## Discussion

QACs are prevalent cationic antimicrobials utilized in antiseptic and disinfectant formulations due to their robust antibacterial activity, which is primarily attributed to interactions with the bacterial cell envelope. However, their practical application is limited by recognized drawbacks, including cytotoxicity and broader safety and environmental concerns. These limitations highlight the necessity for structurally refined QAC derivatives that exhibit enhanced selectivity and performance profiles.

In this study, we investigated the quinuclidin-3-one scaffold as a foundation for developing novel QACs and synthesized a homologous series of long-chained derivatives (QO-C12, QO-C14, QO-C16) to assess structure–activity relationships. The resulting compounds demonstrated high antibacterial potency against clinically significant Gram-positive pathogens, including *Staphylococcus aureus* and *Listeria monocytogenes*. Consistent with established principles governing amphiphilic antimicrobials, alkyl-chain length emerged as a crucial determinant of activity. Among the tested derivatives, QO-C16 exhibited the most potent antibacterial effect, suggesting that increased hydrophobicity enhances efficacy, likely by facilitating more effective membrane interaction and disruption.

A significant finding was the pronounced dependence of MIC values on growth medium. MICs decreased by up to 32-fold in DMEM compared to standard susceptibility testing media. This observation indicates that medium composition can significantly influence the apparent antibacterial potency of QACs. Factors such as ionic strength, protein content, and other medium components may affect compound availability, aggregation behavior, and interactions with bacterial surfaces.

In addition to inhibiting planktonic growth, the QO-QAC series exhibited significant antibiofilm activity against *L. monocytogenes*. Given the intrinsic tolerance of biofilms to antimicrobial agents, this activity is particularly relevant and underscores the potential applicability of these compounds in environments where biofilm formation leads to persistent contamination. Coupled with results from time–kill assays and propidium iodide uptake experiments, which demonstrated rapid and concentration-dependent bactericidal effects, the data consistently support membrane permeabilization as the primary mode of antibacterial action.

Crucially, the biological activity of these derivatives must be weighed against their toxicity profiles. Compared to widely used QACs such as CPC and BAB, the QO-QACs display lower cytotoxicity in healthy human cell lines. However, zebrafish embryo assays revealed a clear correlation between antibacterial potency and developmental toxicity, with increasing alkyl-chain length associated with heightened toxicity. This trend emphasizes the challenge of optimizing QACs—structural features that enhance membrane-disruptive antibacterial activity may also increase interactions with eukaryotic membranes.

In summary, this study identifies quinuclidin-3-one as a promising scaffold for designing QAC-based antimicrobials. The defined structure–activity and structure–toxicity relationships, particularly the central role of alkyl-chain length, provide a rational basis for further structural optimization aimed at improving selectivity while retaining strong antibacterial and antibiofilm performance. Thus, the QO-QAC series offers both mechanistic insight and a foundation for the ongoing development of safer and more effective QAC derivatives.

## Conclusion

Quinuclidin-3-one-based quaternary ammonium compounds exhibit potent antibacterial activity against Gram-positive pathogens, including MRSA. These compounds demonstrate a rapid, membrane-disruptive bactericidal mechanism and significant antibiofilm activity. In comparison to commercial QACs, these derivatives show reduced cytotoxicity and improved selectivity. Overall, the quinuclidin-3-one scaffold represents a promising platform for further optimization toward safer and more effective antimicrobial agents.

## Supplemental data

Supplemental data are available at the following link: https://www.bjbms.org/ojs/index.php/bjbms/article/view/14037/4191.

## Data Availability

The data presented in this study are available on request from the corresponding author.
